# Pb-Pb ages and initial Pb isotopic composition of lunar meteorites: NWA 773 clan, NWA 4734, and Dhofar 287

**DOI:** 10.1111/maps.13547

**Published:** 2020-08

**Authors:** R. E. Merle, A. A. Nemchin, M. J. Whitehouse, J. F. Snape, G. G. Kenny, J. J. Bellucci, J. N. Connelly, M. Bizzarro

**Affiliations:** 1 Swedish Museum of Natural History, SE-104 05 Stockholm, Sweden; 2 School of Earth and Planetary Sciences (EPS), Curtin University, GPO Box U1987, Perth, WA 6845, Australia; 3 Faculty of Earth and Life Sciences, VU Amsterdam, De Boelelaan 1085,1081 HVAmsterdam, the Netherlands; 4 Centre for Star and Planet Formation, GLOBE Institute, University of Copenhagen, Øster Voldgade 5-7, DK-1350 Copenhagen, Denmark

## Abstract

Constraining the duration of magmatic activity on the Moon is essential to understand how the lunar mantle evolved chemically through time. Determining age and initial isotopic compositions of mafic lunar meteorites is a critical step in defining the periods of magmatic activity that occurred during the history of the Moon and to constrain the chemical characteristics of mantle components involved in the sources of the magmas. We have used the in situ Pb-Pb SIMS technique to investigate eight lunar gabbros and basalts, including six meteorites from the Northwest Africa (NWA) 773 clan (NWA 2727, NWA 2700, NWA 3333, NWA 2977, NWA 773, and NWA 3170), NWA 4734, and Dhofar 287A. These samples have been selected as there is no clear agreement on their age and they are all from the dominant low titanium chemical group. We have obtained ages of 2981 ± 12 Ma for NWA 4734 and 3208 ± 22 Ma for Dhofar 287. For the NWA 773 clan, four samples (the fine-grained basalt NWA 2727 and the three gabbros NWA 773, NWA 2977, NWA 3170) out of six yielded isochron-calculated ages that are identical within uncertainties and yielding an average age of 3086 ± 5 Ma. The age obtained for the fine-grained basalt NWA 2700 is not precise enough for comparison with the other samples. The gabbroic sample NWA 3333 yielded an age of 3038 ± 20 Ma suggesting that two distinct magmatic events may be recorded in the meteorites of the NWA 773 clan. The present study aims to identify and assess all potential issues that are associated with different ways to date lunar rocks using U-Pb–based methods. To achieve this, we have compared the new ages with the previously published data set. The entire age data set from lunar mafic meteorites was also screened to identify data showing analytical issues and evidence of resetting and terrestrial contamination. The data set combining the ages of mafic lunar meteorites and Apollo rocks suggests pulses of magmatic activity with two distinct phases between 3950 and 3575 Ma and between 3375 and 3075 Ma with the two phases separated by a gap of approximately 200 Ma. The evolution of the Pb initial ratios of the low-Ti mare basalts between approximately 3400 and 3100 Ma suggests that these rocks were progressively contaminated by a KREEP-like component.

## Introduction

Understanding the early history of the Moon, in particular its mantle–crust differentiation; the timing of magmatic activity; and the potential influence on the latter of large impact events, is critical for refining the existing models of terrestrial planet formation and their evolution during the first billion years of the solar system (e.g., Crawford et al. 2012). As with their terrestrial equivalents, lunar basalts and mafic plutonic rocks are thought to have crystallized from melts derived from the lunar mantle (Basaltic Volcanism Study Project 1981). Therefore, the initial radiogenic isotopic composition of these rocks provides critical constraints on the chemical composition of their mantle source. On Earth, this approach has contributed significantly to attempts to define the chemical characteristics of the mantle and its evolution throughout Earth’s history (e.g., Allegre 1982). However, until recently, the application of a similar methodology on lunar mafic magmatic rocks has been limited by both a small sample set as well as the discrepancy of ages obtained by different radiometric techniques (e.g. Joy and Arai 2013; Snape et al. 2018). The lack of a consensus on their ages precludes the calculation of initial isotopic ratios with the required accuracy to constrain reliable models of lunar mantle evolution. The accuracy of measured isotopic ratios and spatial resolution achieved by the in situ Pb-Pb dating technique using SIMS (secondary ion mass spectrometry) allows determination of (1) isochron ages with a precision better than 1% and (2) precise Pb initial ratios (Snape et al. 2016, 2018, 2019). This results in an opportunity for a systematic comparison of Pb isotopic ratios in lunar rocks.

As lunar meteorites are thought to represent a random sampling of different locations around the Moon (Korotev et al. 2003; Korotev 2005; Warren 2005; Joy and Arai 2013), knowing accurate ages and initial isotope compositions would provide insights into the chemical characteristics of the lunar mantle and its evolution through time at a scale larger than those available from the Apollo samples. Currently available data suggest that the magmatism recorded by mafic lunar meteorites took place between about 4370 and 2650 Ma (Nyquist and Shih 1992; Misawa et al. 1993; Torigoye-Kita et al. 1995; Anand et al. 2003a, 2006; Fernandes et al. 2003, 2009a, 2009b; Nyquist et al. 2005, 2007, 2009; Burgess et al. 2007; Rankenburg et al. 2007; Terada et al. 2007a, 2007b, 2008; Shih et al. 2008; Sokol et al. 2008; Borg et al. 2009; Haloda et al. 2009; Zhang et al. 2010, 2011; Wang et al. 2012; Shaulis et al. 2016, 2017; Snape et al. 2018; Curran et al. 2019). This period of mafic magmatic activity includes, but is significantly wider than, the record provided by the Apollo mafic samples, which is mainly restricted to ages between about 3900 and 3200 Ma (e.g., Head 1976; Nyquist and Shih 1992; Snyder et al. 1997; Joy and Arai 2013; Snape et al. 2016, 2019; Curran et al. 2019). It also partly overlaps estimates obtained from the crater size–frequency distribution, which range from 4000 to 1200 Ma with a major phase of activity between 3800 and 3300 Ma (Hiesinger et al. 2003, 2011). Taken together, these studies suggest that lunar basaltic magmatism lasted from 4370 until ~1500 Ma, with a major phase of continuous activity occurring between 3900 and 3200 Ma, and a possible minor phase at ~2200–1800 Ma (Head and Wilson 1992; Nyquist and Shih 1992; Shearer and Papike 1999; Hiesinger et al. 2003; Basilevsky et al. 2010; Morota et al. 2011; Joy and Arai 2013; Curran et al. 2019).

Nevertheless, the timing of the lunar magmatic activity is still not comprehensively constrained due to the general lack of samples. Furthermore, attempts to date a specific mafic lunar rock using different methods have commonly yielded conflicting ages outside their respective uncertainties. Consequently, it is difficult to compare information provided by different meteorite samples and to correlate meteorite data to the information recorded by the Apollo samples or obtained from the crater size–frequency distribution. This also precludes resolving whether (1) the low-Ti volcanic activity was continuous or not (Haloda et al. 2009) and (2) if the eruption and emplacement of all low-Ti mare basalts occurred after the eruption of high-Ti basalts, as the ages of Apollo and Luna samples indicate (Taylor et al. 1991; Snape et al. 2019). Alternatively, this may be a reflection of sampling bias, as indicated by remote sensing work (Morota et al. 2011).

A further complication is related to uncertainty in the possible pairing of some meteorite samples, in which the individual recovered samples are assumed to have originated from the same mafic body, lava flow, or intrusion and can be considered to be a single sample with unique age and isotopic signature. Existing uncertainties in the age determinations typically preclude confident assessment of possible pairing of different fragments. Therefore, an improved ability to determine ages and isotope systematics of the samples can be used to provide additional ways to establish a similar origin for different meteorites.

In this contribution, we have investigated the Pb isotope systems in the samples Northwest Africa (NWA) 4734, Dhofar 287, and six stones supposedly paired with NWA 773. The reason for this selection is that most of these rocks appear to be among the youngest mafic lunar meteorites (e.g., Curran et al. 2019). However, despite several attempts to date these rocks (and for some of them by several different analytical methods), there is no agreement on their ages. This is particularly evident for the samples from the NWA 773 clan where 5 (NWA 3160, NWA 773, NWA 3170, NWA 6950, and NWA 2977) of 16 stones have been dated so far, yielding ages ranging from 2650 to 3290 Ma (Fernandes et al. 2003; Burgess et al. 2007; Borg et al. 2009; Nyquist et al. 2009; Zhang et al. 2011; Shaulis et al. 2017). Consequently, it is not clear whether the stones of the NWA 773 clan are correctly paired.

All the samples investigated here are different from the classic types of basaltic rocks established using Apollo samples (Neal and Taylor 1992). All these meteorites appear to be low-Ti, but display significant enrichment in incompatible elements, in particular rare earth element (REE) patterns resembling those of the KREEP basalts (typically, Apollo 15 basalts; Fagan et al. 2003; Jolliff et al. 2003; Borg et al. 2009; Zhang et al. 2011; Wang et al. 2012), which have high K, REE, and P contents (e.g., Shearer et al. [2006] and references included). Although the KREEP signature and the hypothetical “urKREEP” (Warren and Wasson 1979) reservoir is thought to have its origins in the final stages of the solidifying lunar magma ocean (LMO), the nature of the connection with KREEP-rich basalts is not clear. The KREEP chemical affinity found in meteorite and Apollo basaltic samples has been attributed to either low-degree partial melting of compositionally heterogeneous late formed LMO cumulates (e.g., Taylor et al. 2012; Elardo et al. 2014) or assimilation of a KREEP material during ascent of the magmas (e.g. Shih 1977; Warren and Wasson 1979; Shearer and Papike 1999; Wang et al. 2012). Therefore, constraining the age and Pb isotope signature of these rocks will also help in deciphering the origin of this KREEP chemical signature.

## Mineralogical, Petrological, and Geochronological Background

### NWA 4734

NWA 4734 has been described as an unbrecciated basalt displaying a coarse-grained texture containing mm-sized crystals of pyroxene (50% vol) and plagioclase (30% vol, Connolly et al. 2008). The texture is subophitic with zoned pyroxene (En_65_Fs_21_Wo_13_ to En2Fs83Wo15) and plagioclase laths (An75-An91, average An_89_) partly transformed into maskelynite. Silica and silica-feldspar glass intergrowths are also present as minor components (7% vol). Impact melt patches also exist. Accessory phases include fayalite (Fa_80-95_), baddeleyite, ilmenite, zirconolite, tranquilityite, pyrrhotite, and metals (7% vol, Connolly et al. 2008). This texture as well as major and trace element bulk-rock and mineral chemistry are very similar to those of the NWA 032/479 and LAP 02205/02224/02226/02436/ 03632/04841 lunar basaltic meteorites (Day et al. 2006; Connolly et al. 2008; Korotev et al. 2009; Fernandes et al. 2009b; Wang et al. 2012; Elardo et al. 2014). Whereas NWA 4734 can be viewed as a low-Ti basalt in the context of Apollo mare basalt samples, it also has the highest recorded incompatible element content of any non-KREEP lunar basalt (Elardo et al. 2014).

NWA 4734 has been dated by several methods, including whole-rock ^40^Ar/^39^Ar dating (2743 ± 64 Ma; Fernandes et al. 2009b); Sm-Nd and Rb-Sr isochrons (3024 ± 27 Ma and 3083 ± 42 Ma, respectively; Elardo et al. 2014); weighted average of ^207^Pb/^206^Pb ages from U-Pb baddeleyite analyses (3073 ± 15 Ma; Wang et al. 2012); and by EMPA U-Pb dating on monazite (3190 ± 190; Jambon and Devidal 2009).

### Dhofar 287A

Dhofar 287 meteorite contains two lithologies that are a low-Ti olivine–pyroxene basalt (Dhofar 287A) comprising 95% of the volume of the recovered stone and a regolith breccia lithology (Dhofar 287B) that represents the remaining 5% of the volume (Anand et al. 2003a). In this contribution, we investigated the basaltic lithology (Dhofar 287A) comprising 1–2 mm high-Mg olivine (Fo_50_-Fo_70_) and subordinate amounts of elongated 0.5 mm-sized pyroxene phenocrysts (En_2-50_Wo_10-25_) embedded in a finer grained matrix of elongated pyroxene and plagioclase (An72-84) as well as chromite, ilmenite, Si-K-rich glass, and phosphate (Anand et al. 2003a). Plagioclase has been transformed into maskelynite (Anand et al. 2003a).

Whole-rock chemistry indicates that Dhofar 287A is an atypical low-Ti basalt with some similarities to the Apollo 12 and 15 samples (Anand et al. 2003a). The high REE contents have been interpreted to reflect a contribution of a KREEP component (Anand et al. 2003a). However, this conclusion has been challenged on the basis that the REE contents, in particular Sm, are not very different from those of Apollo mare basalts (Korotev 2012).

Dhofar 287A has been previously dated at 3460 ± 30 Ma by Sm-Nd isochron (Shih et al. 2002). This isochron is based on a six-point regression with four of the data points being bulk-rock fractions. Two other fractions show evidence of terrestrial contamination (Shih et al. 2002). Contamination was further indicated by an attempt to constrain an Rb-Sr isochron (Shih et al. 2002), which failed to yield a valid regression. A more recent attempt to date this rock was made using in situ U-Pb and Pb-Pb dating by sensitive high resolution ion microprobe (SHRIMP) on phosphates and feldspars that yielded an age of 3350 ± 130 Ma (Terada et al. 2008).

### NWA 773 Clan

The NWA 773 clan consists of 16 stones containing four different lithologies, including regolith breccia, olivine gabbro, ferro-gabbro, and olivine basalt. All these rocks have been the subject of extensive mineralogical, geochemical, and geochronological investigation (e.g., Fagan et al. 2003, 2014; Fernandes et al. 2003; Jolliff et al. 2003; Bunch et al. 2006; Burgess et al. 2007; Zeigler et al. 2007; Weisberg et al. 2008; Borg et al. 2009; North-Valencia et al. 2014; Shaulis et al. 2017; Valencia et al. 2019). The whole-rock geochemistry of the NWA 773 clan is atypical of many other lunar rocks, being more enriched in iron as shown by their mineral compositions but also having major element compositions similar to very low titanium (VLT) rocks (Jolliff et al. 2003). However, contrary to the other VLT volcanic rocks, the NWA 773 clan is enriched in light REE and large ion lithophile elements with a clear KREEP signature suggested by the REE patterns, including a strong negative Eu anomaly (Jolliff et al. 2003). Among the samples investigated here, the NWA 773 clan displays the strongest KREEP chemical signature. The mineralogical, geochemical, and geochronological background of the rocks investigated in the present study is summarized below.

NWA 2700 is a stone containing abundant small olivine gabbro clasts and sparse clasts of fine-grained basalt and regolith breccia. The olivine gabbro contains approximately 50% of olivine (Fa_29-35_), low-Ca and high-Ca clinopyroxene (Fs_22-28_Wo_6-10_ and Fs_13_Wo_38_, respectively), and plagioclase. Accessory minerals are represented by alkali feldspar, Cr-spinel, ilmenite, phosphate, and troilite (Bunch et al. 2006). The basalt clasts contain high-Ca clinopyroxene (Fs_44_Wo_29_ to Fs_58_Wo_23_), plagioclase, and K-Si-rich glass (Bunch et al. 2006). This sample has not been dated previously.

NWA 2727 was recovered as four separate stones and contains four lithologies including basalt as the dominant lithology, olivine gabbro occurring as clasts, ferro-gabbro, and breccia. The basaltic clasts have porphyritic textures containing olivine (Fa_28-99_) and chromite phenocrysts. These minerals are included in a rapidly quenched matrix formed by low-Ca pyroxene, K-feldspar, ilmenite, phosphate, baddeleyite, troilite, and glass (Bunch et al. 2006). The plutonic clasts contain low-Ca pyroxene (Fs_23-31_Wo_9-11_), high-Ca pyroxene (Fs_24-47_Wo_24-32_), anhedral olivine (Fa_34-41_), and plagioclase (An_81-94_). As with NWA 2700, this sample has not been dated previously.

NWA 773 is an impact breccia that was recovered as three stones containing very large clasts of cumulate olivine gabbro (Fagan et al. 2003; Jolliff et al. 2003). Mineral proportions vary significantly in this sample with olivine (~Fo_68_) amounts ranging from 55% to 48%, pigeonite (En_60-67_Wo_6-16_Fs_24-27_), augite (En47-50 Wo_40-33_Fs_13-17_) from 40% to 28%, and plagioclase (An_80_-_90_) from 14% to 11% (Fagan et al. 2003; Jolliff et al. 2003). Clasts of both magnesian and ferroan gabbro have been identified in this sample (Shaulis et al. 2017), which would explain the variations in mineral proportions and chemistry. Moreover, in addition to the olivine cumulate lithology, pyroxene gabbro, symplectite, and alkali-ferroan clasts occur in the breccia (Fagan et al. 2014). The accessory minerals in this sample include K-feldspar, ilmenite, Cr-spinel, phosphate, baddeleyite, troilite, and Fe-Ni metal (Jolliff et al. 2003; Borg et al. 2004; Tartèse et al. 2014). In terms of major element concentrations, the gabbroic olivine cumulate clast has a bulk composition typical of very low-Ti basalts but is enriched in rare earth and large-ion lithophile elements and displays a very strong negative anomaly in Eu that is unusual for the VLT basalts (Fagan et al. 2003; Jolliff et al. 2003). Three previous attempts resulted in a range of ages. The weighted average ^207^Pb/^206^Pb age of 3099 ± 28 Ma and the U-Pb concordia age of 3112 ± 33 Ma were obtained from phosphates from the same sample, containing olivine cumulate and breccia (Shaulis et al. 2017), but both result in a relatively high MSWD (>1.5) and low probability of the fit (*P* < 0.05), indicating a scatter of the data beyond analytical uncertainty. Baddeleyite from the olivine cumulate investigated by the U-Pb method using laser ablation ICP-MS yielded a weighted average ^207^Pb/^206^Pb age of 3129 ± 12 Ma (date calculated using baddeleyites from the magmatic clast; data in Shaulis et al. 2017). The olivine cumulate clast has also been dated at 2993 ± 32 Ma by the Sm- Nd isochron method (Borg et al. 2009). ^40^Ar/^39^Ar measurements of whole-rock fractions of the olivine cumulate produced a disturbed Ar degassing spectrum yielding a mini-plateau age (based on only 54% of degassed ^39^Ar) of 2910 ± 10 Ma (Fernandes et al. 2003).

NWA 2977 is formed entirely of olivine cumulate gabbro that is identical in texture and mineralogy to those in NWA 773 and NWA 2700. It has a cumulate texture containing 51% of olivine (Fa32), 23% of low- Ca clinopyroxene (En_66_Fs_27_Wo_7_), 9% of high-Ca pyroxene (En_55_Fs_16_Wo_29_), and 14% of plagioclase (An_92_), with K-feldspar, chromite, ilmenite, phosphate, and troilite as accessory minerals (Bunch et al. 2006). Several attempts to date this rock include an Rb-Sr mineral and whole-rock isochron-calculated age of 3290 ± 110 Ma (Nyquist et al. 2009), a weighted average of ^207^Pb/^206^Pb ages of 3123 ± 7 Ma from analyses of baddeleyite (Zhang et al. 2011), an Sm-Nd mineral and whole-rock isochron age of 3100 ± 50 Ma (Nyquist et al. 2009), and a whole-rock ^40^Ar/^39^Ar mini plateau age of 2770 ± 40 Ma (Burgess et al. 2007).

NWA 3170 is composed of magnesian gabbro, ferroan gabbro, anorthositic gabbro, and polymict breccia. The mafic clasts formed by olivine (Fo_30-40_), low-Ca pyroxene with a wide range of composition in terms of Ca, Fe, and Mg contents (En13-68Fs21-62Wo9-40), plagioclase (An_90-97_) and ilmenite, rare K-rich feldspar, fayalite, silica, phosphate, and baddeleyite as accessory phases (Ruzicka et al. 2014; Shaulis et al. 2017). This sample has been dated by U-Pb method using laser ablation ICP-MS on baddeleyite extracted from three lithologies that are an Mg-gabbro clast, an Fe-gabbro clast, and the breccia matrix. By combining all the U-Pb baddeleyite measurements made in the mafic clasts and the breccia, Shaulis et al. (2017) obtained a weighted average ^207^Pb/^206^Pb age of 3118 ± 14 Ma. The weighted average age of the baddeleyite grains from the magnesian gabbro clast yielded 3138 ± 38 Ma (3135 ± 30 Ma if the grains from the magnesian and ferro gabbros are used in this calculation). Combining all the U-Pb data obtained from baddeleyite and phosphate grains in Mg- and ferroan gabbros lithologies, Shaulis et al. (2017) also suggested an age of 3116 ± 7 Ma for the NWA 773 clan.

NWA 3333 contains four lithologies including olivine cumulate gabbro, ferro-gabbro, basalt, and breccia matrix. The olivine gabbro is formed by 50% volume of olivine (Fo_68_), low-Ca, and high-Ca clinopyroxene (with average compositions of Fs_63_Wo_15_ and Fs_52_Wo_32_, respectively) representing 35% volume and plagioclase (An_92_) representing 15% volume (Weisberg et al. 2008). There is no previous geochronological data from this sample.

## Data Acquisition and Processing

### Data Acquisition

To determine the mineralogy and identify potential targets for Pb-Pb SIMS analyses, the samples were mapped using backscattered electron imaging and energy dispersive X-ray spectroscopy (EDS). The selected samples were mounted in epoxy resin, polished to flatness, cleaned with analytical grade ethanol, and coated with carbon. Images of the samples were acquired using a Thermo-Fisher Quanta FEG650 scanning electron microscope (SEM) fitted with an Oxford Instruments INCA EDS detector and housed at the Swedish Museum of Natural History (Stockholm). The SEM was operated with a 20 kV acceleration voltage and a working distance of 10 mm.

Following SEM imaging, the carbon coating was removed, the sample mounts cleaned with deionized water and analytical grade ethanol, dried down, and finally coated with a 30 nm layer of gold. The Pb-Pb measurements were made using a CAMECA IMS 1280 ion microprobe at the NordSIMS facility of the Swedish Museum of Natural History. The targets were analyzed with a −13 kV ^16^O^2−^ primary beam (10 kV secondary beam) at a working intensity of ~2–3 nA. The instrument was operated with a 45 eV energy window, 3001 μm field aperture, 75 μm entrance slit, and 400 μm contrast aperture. The resulting spot size was of ~10 μm and the mass resolution (*M*/Δ*M*) of 4830. The samples were sputtered before analysis for 300s using a raster size of 15 × 15 μm. ^204^Pb, ^206^Pb, ^207^Pb, and ^208^Pb isotopes were measured simultaneously with four low-noise ion counting electron multipliers (multicollection mode). Each measurement consisted of 80 cycles (20s integration time and 0.8s wait time per cycle).

The U.S. Geological Survey basaltic glass standard BCR-2G was analyzed after every five or six unknowns to monitor the in-run stability of the instrument. The standard was run using a 6000 μm field aperture and 60s of pre-sputter with a raster of 20 × 20 μm. The measured values of BCR-2G were compared to the accepted values of this standard (Woodhead and Hergt 2000) to calculate a correction factor accounting for mass fractionation bias and inter-detector relative gain calibration (see Standard data file in supporting information). A correction factor was calculated for each analytical session and applied to the unknowns analyzed during the same session. Individual measurements of BCR-2G during each analytical session, average values of this standard for each session, and correction factors are given in supporting information.

Backgrounds were measured before and after every standard or unknown. For each analytical session, the ^204^Pb counts were corrected for the average value of the background on the ^204^Pb mass.

### Data Processing

The data processing and filtering procedure were designed to take into account the analysis of different mineral phases containing both non-radiogenic and radiogenic Pb at very different levels of concentration. The raw data were initially filtered based on analytical quality that involves checking the analytical conditions and inspecting the counting rates and isotopic ratios evolution of all the individual runs. The first step of data quality assessment involves rejecting data with field aperture centering (DTFA-x and DTFA-y) exceeding ± 100 digits, above which level aberrations have been observed to degrade peak flatness. Analyses with ^206^Pb count rates lower than 1 cps (counts per second) had standard errors of >50% and were excluded from the final data set.

The Pb isotope ratios were calculated using an inhouse Excel Add-In, which allows calculation of integrated mean ratios based on total counts (hereafter: integrated means method) as well as mean ratios based on counts for each scan (hereafter: scan-by-scan means method). The integrated means method estimates ratios from the sum of all counts accumulated during a single analysis with the uncertainty calculated using the Poisson counting statistic error (square root of the total number of data, applied to the relative beam stability). The “integrated means” method appears to be more robust when determining ratios involving low- abundance isotopes, such as ^204^Pb, which may have had absent counts in some cycles. However, it was found to underestimate uncertainty of ^207^Pb/^206^Pb ratios. The scan-by-scan means method involves the calculation of mean and uncertainty from the individual ratios determined for each scan. It results in a more realistic uncertainty for ^207^Pb/^206^Pb ratios, but appears to overestimate the uncertainties for the ^204^Pb/^206^Pb. The issue of opting for integrated means method versus scan-by-scan means method is partly resolved by removing analyses with low ^206^Pb counts. Comparison of the two protocols for filtered analyses indicates that they give similar values for all ratios in the majority of analytical spots. However, a limited number of analyses still show significantly different standard error (discrepancy higher than 50%) between the two types of error estimation. This indicates a significant change in counting rate during these analyses, which is clear when count rates for individual analyses are plotted against time. These runs have been recalculated to include only parts of the runs where count rates remain stable. A few remaining analyses that show irregular variations of counting rates throughout the runs were discarded.

While the scan-by-scan means method was adopted for most data reported here, this approach struggles to estimate ^204^Pb/^206^Pb and its uncertainty in the small number of analyses where ^204^Pb was extremely low (close to zero). In these rare cases, ^204^Pb/^206^Pb was estimated using the integrated means method.

### Pb-Pb Isochrons

Pb-Pb isochrons were constrained using the Excel add-in Isoplot4 (Ludwig 2008). In general, the Pb-Pb data sets do not form a linear array of data points in the ^204^Pb/^206^Pb versus ^207^Pb/^206^Pb diagram but plot in a triangular space formed by three Pb components that are initial, radiogenic, and terrestrial Pb (Connelly et al. 2012, 2017). The isochron is defined here as the binary mixture of initial and radiogenic Pb and as such is formed by the set of data located to the left-hand side of the three Pb components’ triangular space in the ^204^Pb/^206^Pb versus ^207^Pb/^206^Pb plot (hereafter “leftmost isochron method”; Connelly et al. 2012; Snape et al. 2016). To construct the Pb-Pb isochron, we used the Isoplot algorithm. The data points located to the right of the true isochron are interpreted to include some terrestrial Pb contamination. These data can be eliminated from the regression by using their weighted residuals (see Ludwig 2003) that have high positive values. Several iterations of data filtering are required to reach the definition of a statistically valid regression. This implies (1) mean square weighted deviation or MSWD that expresses the scatter of the data points relative to the regression, must be lower than 2 and (2) the probability of fit (P), which tests if the only reason for scatter from straight line is the analytical errors assigned to the data points, must be higher than 0.05 (see McIntyre et al. 1966; York 1968; Wendt and Carl 1991; Ludwig 2003 for detailed discussion about these parameters). It should be noted that despite the aim of this approach being to yield low MSWD and P> 0.05, it does not a priori imply that statistically valid regressions yielding accurate and precise ages can always be obtained (see results below).

## Results

The entire Pb-Pb data set for all the investigated samples is available in the Pb-Pb data file in supporting information. A summary of the calculated isochron ages and weighted average ^207^Pb/^206^Pb and ^204^Pb/^206^Pb ratios is provided in Table 1. The analyses were made away from alteration products, cracks in minerals, and impact melt veins.

### NWA 4734

**Fig. 1:**
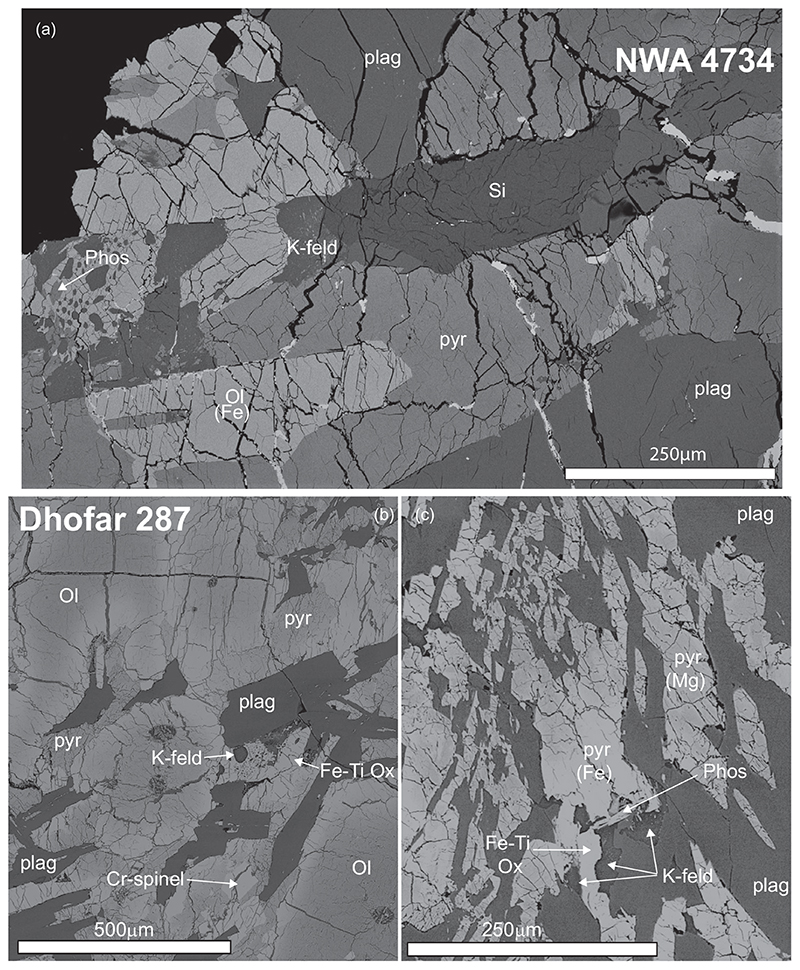
Backscatter electron (BSE) images of representative sections of NWA 4734 and Dhofar 287A. a) ~1 × 0.7 mm image of NWA 4734. b) ~1 × 1 mm image of Dhofar 287A showing the coarse-grained texture dominated by large olivine phenocrystals. c) ~300 × 500 μm image of Dhofar 287A showing a more fined-grained texture of Dhofar 287A with elongated plagioclases arranged as a slightly radial cluster in the left of the image. Also visible is the pyroxene zoning. ol = olivine; CPX = clinopyroxene; Plag = plagioclase; Pyr = pyroxene, Fe-Ti Ox = iron-titanium oxide; K-feld = K-rich feldspar or K-rich feldspathic glass; Phos = phosphate; Sulf = sulfide.

**Fig. 2:**
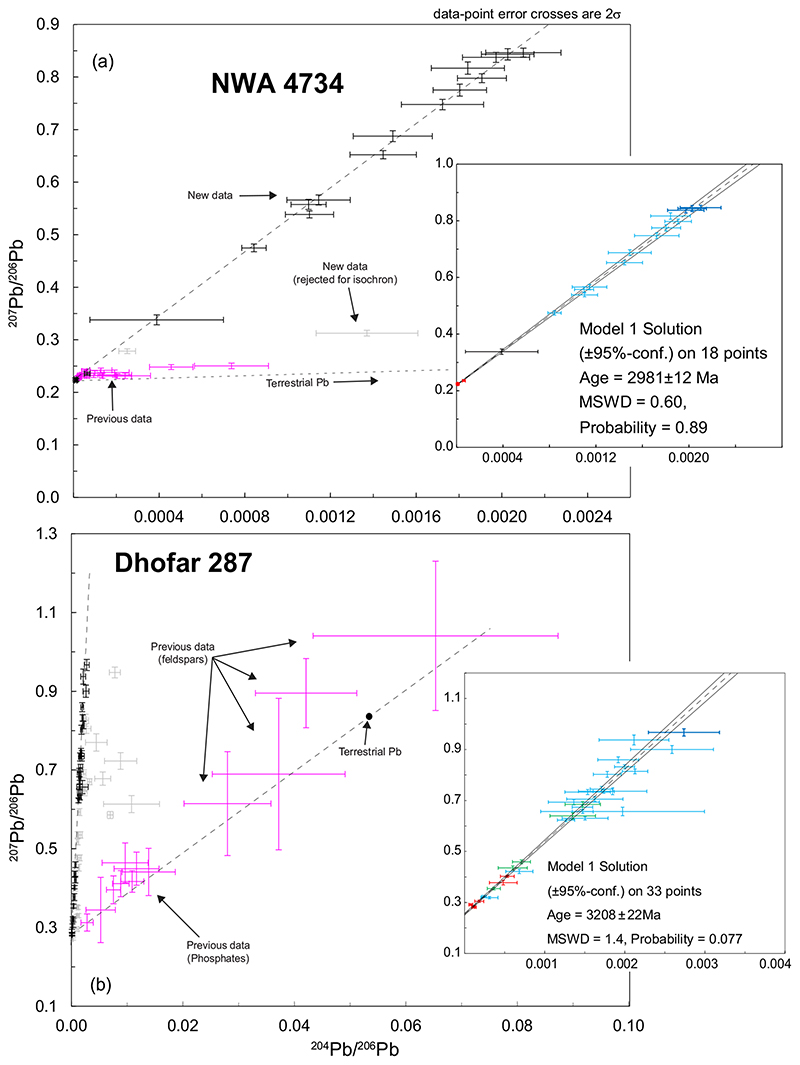
^207^Pb/^206^Pb versus ^204^Pb/^206^Pb plots for (a) NWA 4734 and (b) Dhofar 287 samples. All data points are represented as error crosses. Black crosses are data used for the isochron and light gray crosses are data rejected from the regression. Previously published data are shown as purple crosses in the main plots. Data used for the isochron calculations are shown in the inserts with a color code corresponding to the analyzed phases: red = phosphate; black = sulfide; blue = K-rich feldspar or K-rich feldspathic glass; green = K-rich feldspar + phosphate intricate mixture. The thick blue crosses are data used for calculation of initial ^207^Pb/^206^Pb and ^204^Pb/^206^Pb ratios. Also shown, the composition of average terrestrial Pb using the values from Stacey and Kramers (1975). Decay constants used for age calculation are according to Steiger and Jäger (1977). Previously published data: NWA 4734: Wang et al. (2012). Dhofar 287A: Terada et al. (2008). (Color figure can be viewed at wileyonlinelibrary.com.)

The section of NWA 4734 investigated in this study has a coarse-grained texture that is formed by large clinopyroxene crystals, plagioclase laths, and rare iron-rich olivine grains, titanium-rich ulvöspinel, and iron-titanium oxides (Fig. 1a). Targets for Pb-Pb analyses are identified as phosphates, K-Si rich phases (residual liquid or K-feldspar), and baddeleyite. Twenty spots were analyzed in the samples by SIMS, 18 of which form a robust regression yielding an age of 2981 ± 12 Ma (95% confidence; MWSD = 0.60; P = 0.89, Fig. 2). Three K- feldspar analyses display the highest measured ^207^Pb/^206^Pb ratios and are interpreted to represent the initial Pb composition. These analyses are statistically undistinguishable, yielding weighted averages of ^207^Pb/^206^Pb = 0.8425 ± 0.0055 (2σ, MSWD = 1.02, P = 0.36) and ^204^Pb/^206^Pb = 0.002028 ± 0.000085 (2σ, MSWD = 0.59, P = 0.56, Fig. A1 in supporting information).

### Dhofar 287A

Dhofar 287A also has a coarse-grained texture defined by large (~500 μm) zoned olivine phenocrysts embedded in a matrix composed of plagioclase laths and interstitial pyroxene with minor Cr-spinel, Fe-Ti oxides, and K-feldspar grains (Figs. 1c and 1d). Locally the texture of this sample is finer grained, dominated by elongated plagioclase forming radial clusters and more Fe-rich pyroxene (Fig. 1d). Pb-Pb analytical targets were identified as phosphates, sulfides, and K-Si rich phases scattered in the matrix. Sixty-eight analyses were made in the sample with 33 analyses of K-feldspar or K-feldspar-rich residual glass, phosphates, and intricate mixtures of phosphate and K-feldspar defining a regression that yields an age of 3208 ± 22 Ma (95% confidence; MWSD = 1.4; *P* = 0.077; Fig. 2). The highest ^207^Pb/^206^Pb was measured in a K-feldspar grain and is considered to represent a minimum estimate of the initial Pb composition for Dhofar 287A (^207^Pb/^206^Pb = 0.9669 ± 0.0143; ^204^Pb/^206^Pb = 0.002739 ± 0.000443, 2σ).

### NWA 773 Clan

Six samples of the NWA 773 clan were investigated in this study. Among them, two have a fine-grained texture (basalts NWA 2700 and NWA 2727), three have a coarse-grained texture (gabbros NWA 773, NWA 2977, and NWA 3170). The last sample, NWA 3333, displays both basaltic and gabbroic lithologies, with only the latter investigated for Pb-Pb systematics.

#### Basaltic Samples

##### NWA 2700

**Fig. 3:**
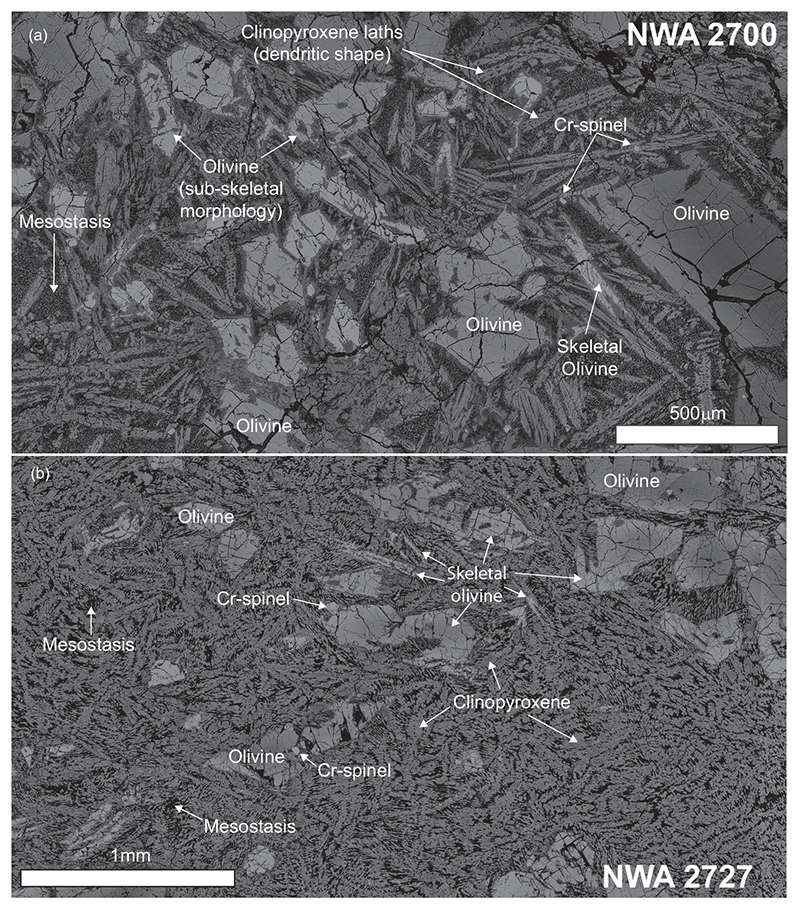
Backscatter electron (BSE) images of representative sections of basaltic samples from NWA 773 clan: (a) NWA 2700 and (b) NWA 2727.

**Fig. 4:**
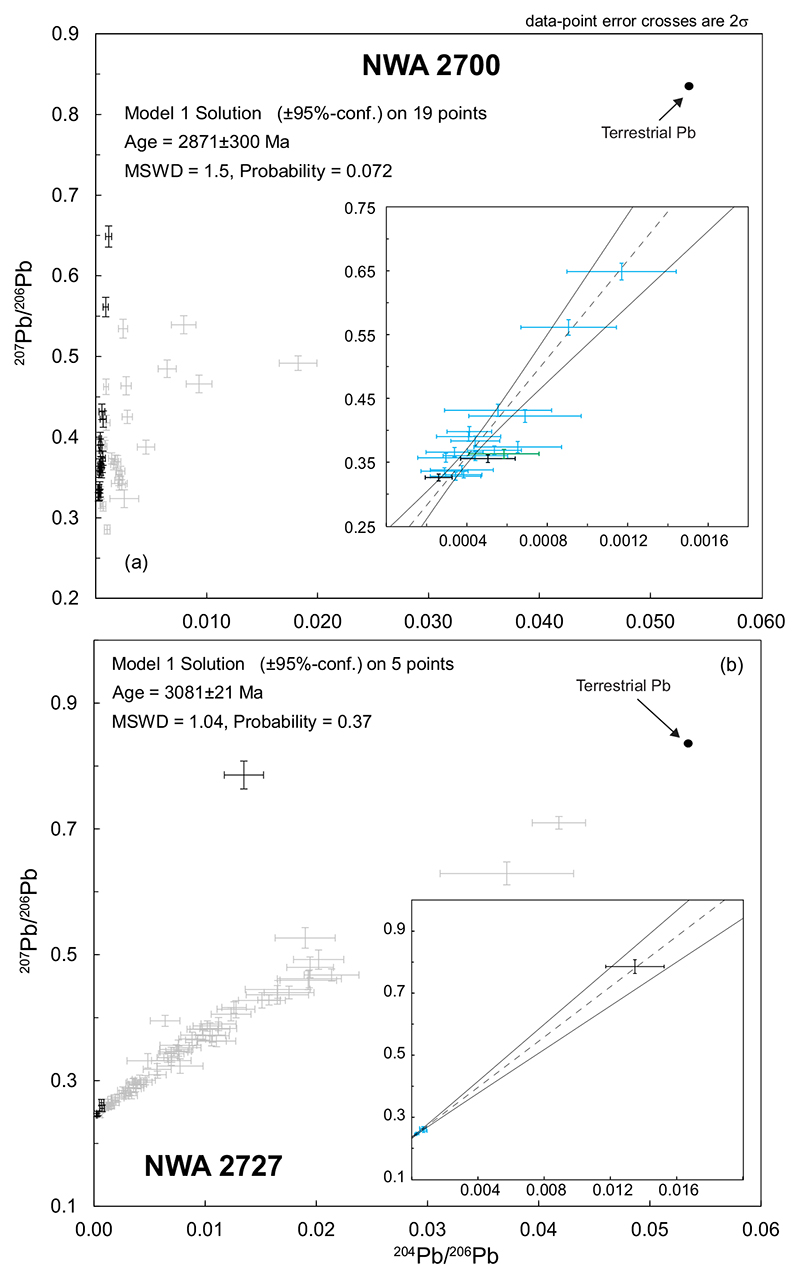
^207^Pb/^206^Pb versus ^204^Pb/^206^Pb plots for basaltic samples of NWA 773 clan. a) Plot showing all the data points obtained by SIMS in the NWA 2700 sample. Also shown, the composition of average terrestrial Pb using the values from Stacey and Kramers (1975). The data rejected from the regression are shown in gray and those kept for the construction of the isochron are shown in black. In insert, the isochron using the same data set. Color-coded error crosses correspond to the local composition of the analyzed mesostasis: blue = K-rich; black = S-rich; red = P-rich; green = K-, P-, and S-poor material. b) ^207^Pb/^206^Pb versus ^204^Pb/^206^Pb plot showing all the data points obtained by SIMS in the NWA 2727 sample. The data rejected from the regression are shown in gray while the data used to build the isochron are shown as black error crosses and the rejected data as gray crosses. In insert, the data used for the construction of the isochron as shown with a color code corresponding to the local composition of the analyzed mesostasis: blue = K-rich; black = S-rich material. Decay constants used for age calculation are according to Steiger and Jäger (1977). Also shown, the composition of average terrestrial Pb using the values from Stacey and Kramers (1975). (Color figure can be viewed at wileyonlinelibrary.com.)

The analyzed piece of NWA 2700 has a porphyritic intersertal texture with 250–600 μm long olivine phenocrysts, with some skeletal crystals (Fig. 3). The groundmass is formed by a network of ~200 μm long clinopyroxene laths with dendritic shapes and scattered Cr-rich spinel. An Si-Al rich cryptocrystalline mesostasis locally containing patches of K-rich and rare P- and S- rich materials is present between rock-forming minerals. These patches were targeted for Pb isotopic investigation with 45 measurements of the mesostasis. Nineteen data points yielded a statistically valid, but very imprecise age of 2871 ± 300 Ma (MSWD = 1.5; *P* = 0.072; Fig. 4a). None of the analytical points are considered to represent an initial Pb composition.

##### NWA 2727

The sample of NWA 2727 investigated here is a basalt with a texture and mineralogy very similar to that of NWA 2700. It has a porphyritic texture with large phenocrysts of olivine (200–700 μm long) embedded in a groundmass of clinopyroxene, rare Cr-rich spinels, and crypto-crystalliner mesostasis located between clinopyroxene (Fig. 3). Compared to NWA2700, the olivine phenocrysts have more pronounced skeletal morphologies whereas the clinopyroxenes in the groundmass do not show a dendritic morphology and the lath shape is less developed. The volume between the clinopyroxenes occupied by the cryptocrystalline mesostasis is smaller than that in sample NWA 2700 (Fig. 3). The cryptocrystalline mesostasis is dendritic and locally contains patches enriched in K, P, and S, which were targeted for Pb isotope analysis.

A total of 76 analyses were made in the mesostasis. The majority of the data show a clear trend toward the composition of terrestrial Pb (Fig. 4b). Nevertheless, five data points define a regression corresponding to an age of 3081 ± 21 Ma (MSWD = 1.04; *P* = 0.37, Fig. 4). The highest ^207^Pb/^206^Pb ratio obtained in this sample from a single analysis of an S-rich patch in the mesostasis is 0.78572 ± 0.0110786 (2σ) corresponding to a ^204^Pb/^206^Pb ratio of 0.01348 ± 0.00088294 (2σ).

#### Gabbroic Samples

**Fig. 5:**
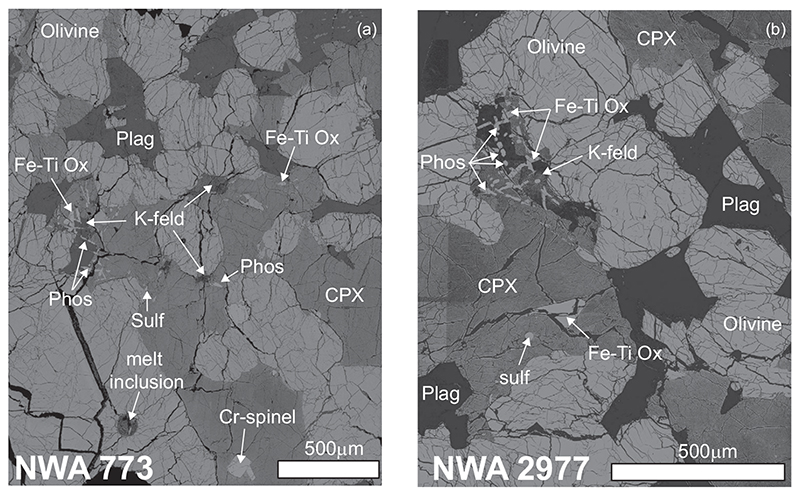
Backscatter electron (BSE) images of representative sections of gabbroic samples from NWA 773 clan: (a) NWA 773 and (b) NWA 2977. CPX = clinopyroxene; Fe-Ti Ox = iron-titanium oxide; K-feld = K-rich feldspar or K-rich feldspathic glass; Phos = phosphate; Sulf = sulfide.

The gabbroic samples have a coarse-grained texture formed by large (>250 μm) subhedral crystals of olivine (50% vol), interstitial clinopyroxene (30% vol.), and plagioclase (15% vol) and, in decreasing order of abundance, Cr-spinels, phosphates, Fe-Ti oxides, K-rich feldspars or K-rich feldspathic glass, sulfides, and Zr-rich phases (Fig. 5). The phosphates are typically closely associated with the K-rich phase and show an elongated shape (Fig. 5). It should be noted that melt inclusion pockets occur within olivine phenocrysts (Fig. 5).

##### NWA 773

**Fig. 6:**
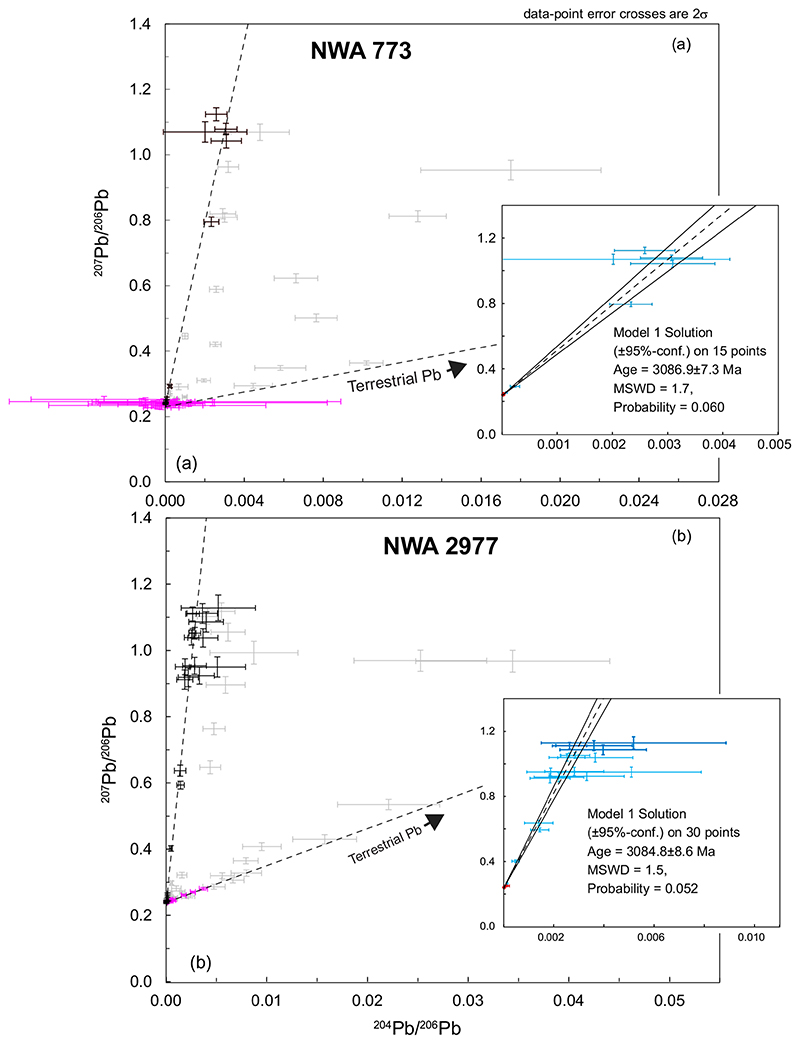
^207^Pb/^206^Pb versus ^204^Pb/^206^Pb plots for gabbroic samples of NWA 773 clan. a) NWA773 data. The data rejected for the construction of the isochron are shown in light gray while those used for the isochron are shown in black. Previously published data are shown in purple (data from Shaulis et al. 2017). In insert, the isochron with the color-coded data according to the analyzed phase. Same color code as in Fig. 2. b) NWA 2977 data. Rejected data: light gray crosses. Data used for the isochron: black crosses. Previously published data shown in purple (Zhang et al. 2011). In insert: isochron showing color-coded data according to the analyzed phase. Same color code as in (a). The thick blue crosses are data used for calculation of initial ^207^Pb/^206^Pb and ^204^Pb/^206^Pb ratios. (Color figure can be viewed at wileyonlinelibrary.com.)

A total of 57 analyses were made in this sample targeting Zr-, K-, and P-rich phases, of which 15 data points form a regression yielding an isochron age of 3086.9 ± 7.3 Ma (MSWD = 1.7; *P =* 0.06; Fig. 6). In a ^207^Pb/^206^Pb versus ^204^Pb/^206^Pb plot, four analyses of K- rich feldspar form a cluster corresponding to the highest ^207^Pb/^206^Pb ratios measured in this sample. No statistically valid (MSWD < 2, *P>* 0.05) weighted average value could be calculated using the ^207^Pb/^206^Pb ratios of these data, but the highest ^207^Pb/^206^Pb ratio obtained from this sample is 1.124 ± 0.00994 (2σ). However, a weighted average value of the ^204^Pb/^206^Pb ratio can be calculated using these four K-feldspar data and is 0.00286 ± 0.00034 (2σ; MSWD = 0.85; *P =* 0.47; see Fig. A2 in supporting information).

##### NWA 2977

Of 68 analyses of this sample made in phosphates and K-rich feldspars, 30 data points form a robust isochron yielding an age of 3084.8 ± 8.6 Ma (MSWD = 1.5; *P =* 0.052; Fig. 6). The initial isotopic Pb composition was extracted from the weighted average of a cluster of the four K-rich phases showing the highest ^207^Pb/^206^Pb ratios: ^207^Pb/^206^Pb = 1.108 ± 0.014 (MSWD = 1.10; *P* = 0.35, *N* = 4, 2σ), ^204^Pb/^206^Pb = 0.00298 ± 0.00058 (MSWD = 1.5; *P* = 0.21, *N* = 4, 2σ; Fig. A3 in supporting information).

##### NWA 3170

**Fig. 7:**
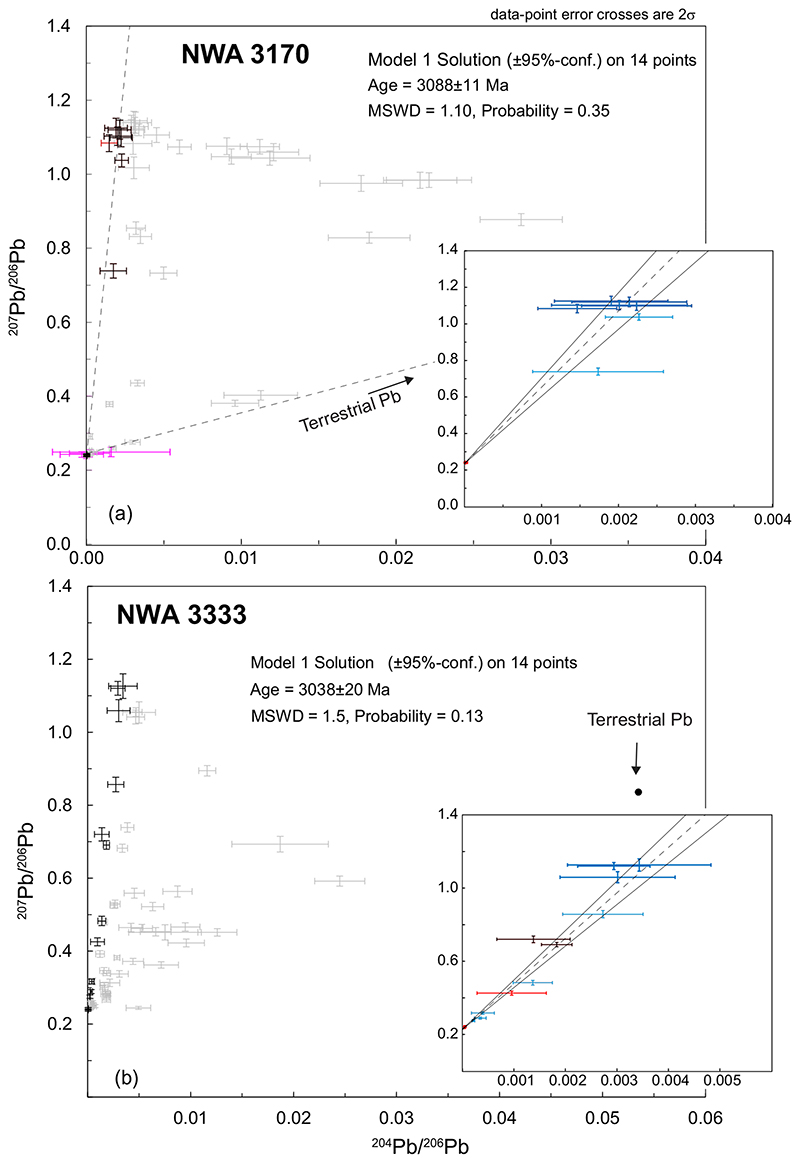
^207^Pb/^206^Pb versus ^204^Pb/^206^Pb plots for gabbroic samples of NWA 773 clan. a) NWA3170 data. The data rejected for the construction of the isochron are shown in light gray while those used for the isochron are shown in black. Previously published data from magmatic lithologies are shown in purple (data from Shaulis et al. 2017). In insert, the isochron with the color-coded data according to the analyzed phase. Same color code as in Fig. 2. The thick blue crosses are data used for calculation of initial ^207^Pb/^206^Pb and ^204^Pb/^206^Pb ratios. b) NWA 3333 data. Rejected data: light gray crosses. Data used for the isochron: black crosses. In insert: isochron showing color-coded data according to the analyzed phase. Same color code as in Fig. 2. The thick blue crosses are data used for calculation of initial ^207^Pb/^206^Pb and ^204^Pb/^206^Pb ratios. (Color figure can be viewed at wileyonlinelibrary.com.)

Sixty-four analyses of this sample were made in K- rich feldspars, phosphates, and sulfides with 14 data points forming a statistically valid isochron corresponding to an age of 3088 ± 11 Ma (MSWD = 1.10; *P* = 0.35; Fig. 7). The best estimate for the initial Pb isotopic composition of the sample was obtained from a cluster of five K-rich phases showing the highest ^207^Pb/^206^Pb ratios, which provided a weighted average composition: ^207^Pb/^206^Pb = 1.105 ± 0.011 (MSWD = 1.8; *P* = 0.13, *N* = 5, 2σ), ^204^Pb/^206^Pb = 0.00186 ± 0.00031 (MSWD = 1.04; *P* = 0.38, *N* = 5, 2σ; Fig. A4 in supporting information).

##### NWA 3333

A total of 70 analyses of this sample were made in sulfides, phosphates, K-rich feldspars, and Zr-rich phases. Of these, 14 data points form a statistically valid isochron corresponding to an age of 3038 ± 20 Ma (MSWD = 1.5; *P =* 0.13; Fig. 7). The best estimate for the initial Pb isotopic composition of the sample was calculated as the weighted average of a cluster of three K-rich phases showing the highest ^207^Pb/^206^Pb ratios: ^207^Pb/^206^Pb = 1.122 ± 0.016 (MSWD = 0.101; *P* = 0.75, *N* = 2, 2σ), ^204^Pb/^206^Pb = 0.00303 ± 0.00055 (MSWD = 0.20; *P* = 0.82, *N* = 3, 2σ; Fig. A5 in supporting information).

## Discussion

Seven of eight samples investigated yielded robust ages, and for five of them, we were able to obtain precise Pb initial isotopic ratios. Our new ages have been obtained by in situ technique from magmatic phases using a proven methodological approach that excludes any obvious products of post-crystallization processes such as alteration phases and impact melts. As a consequence, our new ages are most likely crystallization ages. We note that our new dates are among the youngest dates obtained for NWA 4734, Dhofar 287A, and the NWA 773 clan regardless of the analytical technique. Specifically, our new dates tend to be younger than most of the previously published dates obtained by U-Pb or Pb-Pb methods, which account for almost half of the published ages.

### Comparison with Previously Published Data and Significance of the New Ages

#### Comparison with Previously Published U-Pb and Pb- Pb Data

The discrepancy between U-Pb ages obtained on the basis of analyses of individual grains of U-bearing phases and those determined here from Pb-Pb isochrons stems from several factors including:

1. Accuracy and precision of the technique used to acquire data,
2. Underestimation of effects of post-crystallization processes (resetting, terrestrial contamination) when selecting the data for age calculation,
3. Relatively small errors of mean ages, resulting from pooling multiple analyses,
4. Different approaches to correct for non-in situ accumulated Pb (i.e., combination of lunar initial Pb and terrestrial contamination).

Most in situ analyses of U-bearing minerals are commonly corrected for non-radiogenic Pb, assuming that this non-radiogenic Pb is terrestrial contamination. In contrast, several studies chose not to correct the measured Pb compositions for non-radiogenic Pb, arguing that their ^204^Pb does not exceed background implying the absence of non-radiogenic Pb, and therefore, no correction is necessary (e.g., Shaulis et al. 2017). However, the presence of initial lunar Pb in U- bearing minerals cannot be completely ruled out. More importantly, due to the extremely radiogenic nature of lunar Pb, uncorrected data or data corrected only for terrestrial Pb contamination can result in significant overestimations of the sample ages, even if the amount of initial lunar Pb is very small. In contrast, our approach involves filtering data points inferred to contain contamination Pb until a statistically valid isochron is defined by the leftmost side of a mixing triangle in ^207^Pb/^206^Pb versus ^204^Pb/^206^Pb coordinates as explained above (see the Pb-Pb Isochrons section). A weakness of this approach is that the most radiogenic Pb analyses used to constrain the lower end of the isochron may still contain small proportions of unrecognized terrestrial contamination. In this case, the intercept of the ^207^Pb/^206^Pb axis will underestimate the age of the sample. This means that data not corrected for any terrestrial contamination provide maximum age of a sample, whereas our isochron approach defines a minimum age.

In the following section, data sets from this and previously published studies are discussed to identify and assess potential issues with all kinds of U-Pb and Pb-Pb data. Our present study is therefore an attempt to discuss all issues that are associated with different ways to date lunar rocks using U-Pb–based methods.

##### NWA 4734

NWA 4734 has been dated using electron microprobe analysis of monazite grains and yielded an age of 3190 ± 190 Ma (2σ; Jambon and Devidal 2009) that is too imprecise for comparison as it includes the majority of other published data for this sample within uncertainties.

**Fig. 8:**
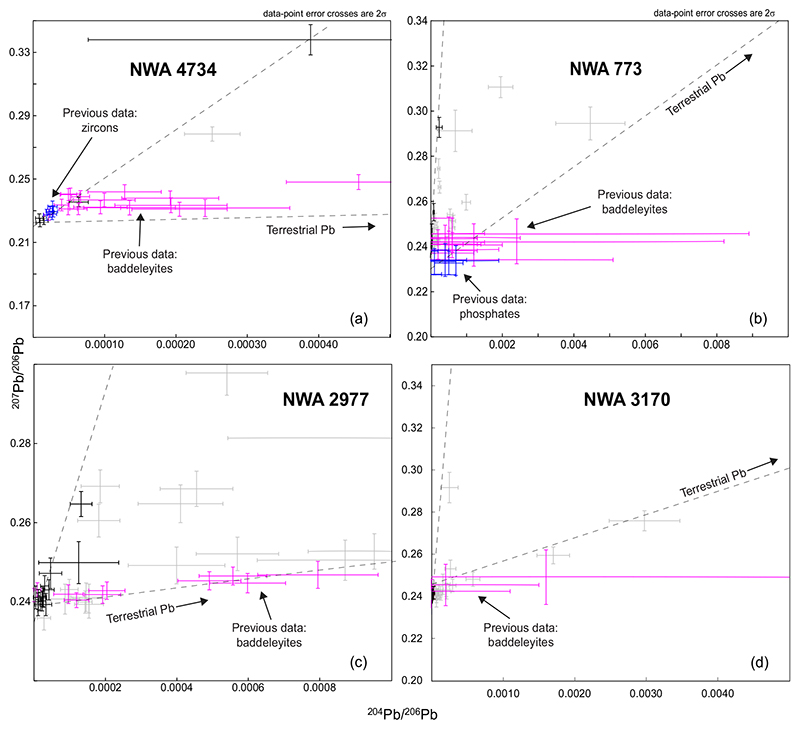
Zoom of ^207^Pb/^206^Pb versus ^204^Pb/^206^Pb diagram on the most radiogenic data for comparing the previously published data from NWA 4734 (a), NWA 773 (b), NWA 2977 (c), and NWA 3170 (d) with our new data. For each sample, all the data are plotted together and the previously published data are shown in purple for baddeleyites and blue for zircons or phosphates. For NWA 773 (b) and NWA 3170 (d), only baddeleyite and phosphate grains found in the magmatic lithologies are shown. The new data used for the isochron are shown in black and the new data rejected for isochron in light gray. Previously published data: NWA 4734: Wang et al. (2012); NWA 3170 and NWA 773: Shaulis et al. (2017), and NWA 2977: Zhang et al. (2011). (Color figure can be viewed at wileyonlinelibrary.com.)

The weighted average ^207^Pb/^206^Pb age of 3073 ± 15 Ma was calculated based on in situ U-Pb analyses of zircon and baddeleyite grains (Wang et al. 2012) and is older than the age of 2981 ± 12 Ma determined here using the Pb-Pb isochron method. The age determined by Wang et al. (2012) assumes that all ^204^Pb has been derived from terrestrial contamination and was corrected using Stacey and Kramers’s (1975) model describing evolution of terrestrial Pb. A large number of baddeleyite analyses appear to trend toward Stacey and Kramers’s (1975) model Pb (Fig. 8a), suggesting significant contamination by terrestrial Pb. However, these analyses can also contain small amounts of unrecognized lunar initial Pb. This possibility is supported by the location of all zircon analyses and four of the most radiogenic baddeleyite analyses from Wang et al. (2012) on, or very close to, the Pb-Pb isochron defined here, but slightly above the most radiogenic phosphate analyses included in the isochron calculation. Constraining a Pb-Pb isochron from zircon and baddeleyite analyses of Wang et al. (2012), and using the approach adopted for our data, results in an age of 2997 ± 36 Ma (95% confidence, MSWD = 1.02, *P* = 0.4, *N* = 7; Fig A7 in supporting information), indistinguishable from the 2981 ± 12 Ma age proposed here.

##### Dhofar 287A

The isochron constrained from phosphate and plagioclase analyses by SHRIMP defined an age of 3350 ± 130 Ma (Terada et al. 2008). The feldspar data display a clear trend toward the modern terrestrial Pb composition (Fig. 2) of Stacey and Kramers (1975) suggesting significant contamination by terrestrial Pb of the analyzed feldspars. Nevertheless, the age obtained by Terada et al. (2008) is indistinguishable within uncertainties from the more precise age presented here of 3208 ± 22 Ma (Fig. 2).

##### NWA 773 Clan

Regardless of differences in uncertainty related to the analytical method (LA-ICP-MS versus SIMS), baddeleyite in samples from NWA 773 clan gave very similar ^207^Pb/^206^Pb ages of 3129 ± 12 Ma for NWA 773 (ICP-MS data; Shaulis et al. 2017); 3123 ± 7 Ma for NWA 2977 (SIMS data; Zhang et al. 2011); and 3138 ± 38 Ma for NWA 3170 (ICP-MS data; same magnesian gabbro lithology investigated here; see Shaulis et al. 2017). These ages are also about 30–35 Ma older than those obtained for the same samples using the isochron method in our study. Nevertheless, the data of Shaulis et al. (2017) are clustered around the most radiogenic analyses obtained here and do not appear to be very different from those when plotted on ^207^Pb/^206^Pb versus ^204^Pb/^206^Pb when uncertainties are taken into account (Figs. 8b and 8d). By contrast, the Zhang et al. (2011) analyses plot along the line between the modern Stacey and Kramers (1975) terrestrial Pb composition and the most radiogenic analyses from this study (Fig. 8c), suggesting that the only difference between our results and those of Zhang et al. (2011) is the presence of a larger proportion of terrestrial Pb contamination in the latter. In addition, phosphate and baddeleyite data presented by Shaulis et al. (2017) for NWA 773 define a U-Pb age of 3094 ± 11 Ma (upper intercept on a concordia plot; 95% confidence; *N* = 26, MSWD = 1.3; P = 0.17). The baddeleyite age presented by Shaulis et al. (2017) for NWA 3170 yield an isochron age of 3135 ± 39 Ma (95% confidence, *N =* 4, MSWD = 0.25; P = 0.78) when data are processed using the same approach we used for our samples. This date is indistinguishable from our new age.

#### Comparison with Previously Published ^40^Ar/^39^Ar, Sm- Nd, and Rb-Sr data

**Fig. 9:**
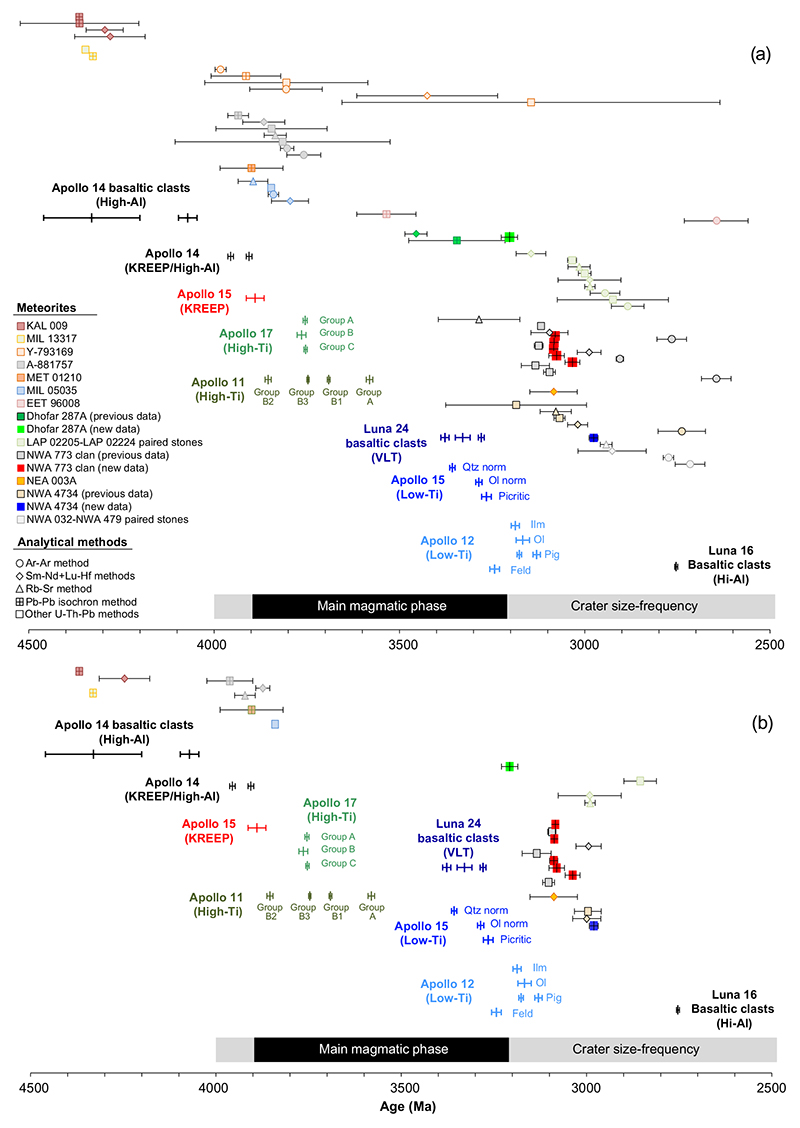
Compilation of ages from gabbroic and basaltic lunar meteorites and Apollo mafic rocks. Analytical methods are also shown. All uncertainties are at 2σ level. a) Compilation of all previously published ages as for lunar gabbroic and basaltic meteorites as well as new ages of NWA 4734, Dhofar 287A, and NWA 773 clan samples obtained in this study. b) Filtered age data set for lunar mafic meteorites. In both plots, the ages of Apollo mafic samples (compilation from Snape et al. 2019) as well as the span of magmatic activity as inferred from crater size-frequency distribution (Hiesinger et al. 2003, 2011) are shown. Data for KAL 009 from Snape et al. (2018), Terada et al. (2007), Sokol et al. (2008), and Shih et al. (2002). Data for MIL 13317 from Shaulis et al. (2016) and Snape et al. (2018). Data for Y-793169 from Torigoye-Kita et al. (1995). Data from A-881757 from Fernandes et al. (2009a, 2009b) and Misawa et al. (1993). Data for MET 01210 from Terada et al. (2007b). Data for MIL 05035 from Zhang et al. (2010) and Fernandes et al. (2009a, 2009b) and Nyquist et al. (2007). Data for EET 96008 from Fernandes et al. (2009a, 2009b) and Anand et al. (2003b). Data for Dhofar 287A from this study, Terada et al. (2008), and Shih et al. (2002). Data for LAP 02224/LAP 02205 clan from Wang et al. (2012), Zhang et al. (2010), Fernandes et al. (2009a, 2009b), Rankenburg et al. (2007), Anand et al. (2006), and Nyquist et al. (2005). Data for NWA773 clan from this study, Shaulis et al. (2017), Borg et al. (2009), Burgess et al. (2007), Nyquist et al. (2007), Fernandes et al. (2003). Data for NEA 003A from Haloda et al. (2009). Data for NWA 4734 from this study, Elardo et al. (2014), Wang et al. (2012), Fernandes et al. (2009a, 2009b) and Jambon and Devidal (2009). Data for NWA 032-NWA 479 from Borg et al. (2009) and Fernandes et al. (2003, 2006). Data for basaltic clasts in sample 14321 (Apollo 14) from Dash et al. (1987), Mark et al. (1974), and Compston et al. (1971, 1972). Data for Luna 16 and 24 basaltic clasts from Cohen et al. (2001). The compilation of the ages from the lunar basaltic meteorites is available in supporting information. This compilation includes the published ages, notes regarding data quality assessment and filtering procedure, as well as recalculated ages when the data were reprocessed. (Color figure can be viewed at wileyonlinelibrary.com.)

Nine ages were previously obtained using the ^40^Ar/^39^Ar, Sm-Nd, and Rb-Sr methods for the same samples investigated in this study. Three ^40^Ar/^39^Ar ages obtained for NWA 2977, NWA 773, and NWA 4734 are similar (2770 ± 40, 2779 ± 14, and 2743 ± 64 Ma, respectively; Fernandes et al. 2003, Burgess et al. 2007; Fernandes et al. 2009b). However, these ages are significantly younger that those determined from other systems (Fig. 9), including isochron Pb-Pb ages obtained in this study (NWA 2977: 3085 ± 9 Ma, NWA 773: 3088 ± 7 Ma; NWA 4734: 2981 ± 12 Ma). The ^40^Ar/^39^Ar system is prone to resetting at relatively low temperatures (potentially during impacts) when compared to other isotope systems, which can result in disturbed ^39^Ar release patterns (e.g., Jourdan 2012). The Ar degassing patterns of NWA2977, NWA 773, and NWA 4734 display such disturbance, and the ages of these samples were calculated from so-called ^40^Ar/^39^Ar mini-plateaus involving approximately 50% of total degassed Ar (Fernandes et al. 2003, 2009a, 2009b; Burgess et al. 2007), which should be considered with caution (e.g., Olierook et al. 2017). Consequently, it is possible that the discrepancy between the new Pb-Pb ages and the 2.7–2.8 Ga age determined using the ^40^Ar/^39^Ar method reflects a reheating event that affected all studied meteorite samples and post-dates their formation.

Two samples NWA2977 and NWA 4734 were analyzed using the Rb-Sr method (Nyquist et al. 2009; Elardo et al. 2014), although only a two-point isochron was constrained for the latter. Both ages (3290 ± 100 and 3083 ± 42, respectively) are significantly older than our new ages obtained from Pb-Pb isochrons. Nevertheless, these Rb-Sr ages are also older than Sm- Nd ages (NWA 2977: 3100 ± 50 Ma, Nyquist et al., 2009; NWA 4734: 3024 ± 27, Elardo et al. 2014), suggesting that the Rb-Sr system in these meteorites has been disturbed, probably resulting in partial Rb loss and decrease of Rb-Sr ratios.

Three of four Sm-Nd ages obtained for Dhofar 287A (3460 ± 30 Ma; Shih et al. 2002), NWA773 (2993 ± 32 Ma; Borg et al. 2009), and NWA4734 (3024 ± 27 Ma; Elardo et al. 2014) also differ significantly from our new Pb-Pb ages (Dhofar 287A: 3208 ± 22 Ma; NWA 773: 3088 ± 7 Ma), while the age obtained for NWA2977 (3100 ± 50 Ma; Nyquist et al. 2009) is similar within the uncertainties. The Sm-Nd age of 3024 ± 27 Ma for NWA4734 (Elardo et al. 2014) is older than our new Pb-Pb age by only 3 Myr. The Sm- Nd ages, obtained for the two samples NWA 773 and NWA 2977 from the NWA773 clan (Borg et al. 2009; Nyquist et al. 2009), appear to be different from each other, while our new Pb-Pb ages of these samples are similar within small uncertainties of 7–9 Ma. This indicates possible redistribution of Sm and Nd on the scale of the analyzed samples.

### Implication for the Timing of Lunar Magmatism

#### New Age of the NWA 773 Clan

The calculated isochron ages of the gabbros NWA 773, NWA 2977, and NWA 3170 are close to 3086 Ma and identical within uncertainties that do not exceed 1%. The age of NWA 2700 has an uncertainty of 10%, which we consider too large for a meaningful interpretation. The age of the fine-grained basalt NWA 2727 is indistinguishable from the ages of investigated gabbro samples (NWA 773, NWA 3170, NWA 2977). The similarity of these ages (excluding the imprecise age obtained for NWA 2700) confirms that they formed at the same time. Combining the four data sets yields a weighted average age of 3086.1 ± 4.8 Ma (MSWD = 0.16; P = 0.92). Also belonging to the NWA 773 clan, the stone NWA 6950 was dated by LA-ICP- MS analysis of baddeleyites at 3100 ± 16 Ma (Shaulis et al. 2017), which is identical to the average age of NWA 773 clan calculated here within uncertainty.

NWA 3333 yielded a statistically valid age of 3038 ± 20 Ma from a 14-point regression. This age is younger than the other samples of the NWA 773 clan that we investigated. From a methodological point of view, there is no evidence to relate the observed age difference between NWA 3333 and the other stones of the NWA 773 clan to an effect of terrestrial Pb contamination. This sample contains abundant large magnesian olivine grains, consistent with an Mg-gabbro lithology (Shaulis et al. 2017). Many stones of the NWA 773 clan include several clasts of different lithologies as documented in NWA 2727 and NWA 3170. As such, the large range of mineral proportions in the Mg-gabbro clasts in NWA 773 could reflect different rocks. All NWA 773 clan samples are not necessarily related to a single fall but rather to the same source locality on the Moon (Calzada-Diaz et al. 2015). Therefore, it is possible that this area encompasses several magmatic units as suspected based on petrological data (e.g., Fagan et al. 2003) but having different ages. Our new age of 3038 ± 20 Ma for NWA 3333 seems to confirm the date of 2995 ± 34 Ma obtained by Sm-Nd isochron in a clast of NWA 773 (Borg et al. 2009). This observation suggests the presence of samples from at least two magmatic events in the Mg-gabbros of the NWA773 clan. Alternatively, this sample might have a completely different origin, even though showing chemical and mineralogical characteristics similar to other samples from NWA773 clan.

#### Toward a New Timing of the Magmatic Activity on the Moon

The results from crater size–frequency distribution investigations and previous age compilations of lunar basalts suggest that (1) mafic magmatic activity appears to be continuous from 4000 Ma until 1200 Ma; (2) most of the mare basalts would have been emplaced between 3800 and 3200 Ma; (3) a peak of basaltic magmatism (mare basalts effusion) occurred between 3800 and 3600; and (4) after this peak, the effusive activity waned progressively until 2000 Ma (e.g., Head 1976; Schultz and Spudis 1983; Head and Wilson 1992; Nyquist and Shih 1992; Shearer and Papike 1999; Hiesinger et al. 2003, 2011; Shearer et al. 2006; Anand and Terada 2008; Basilevsky et al. 2010; Joy and Arai 2013; Curran et al. 2019; Tartèse et al. 2019).

The new age of Dhofar 287A falls within the range of Apollo 12 low-Ti basalts of about 3250–3100 Ma. However, time constraints for NWA4734 and the NWA 773 clan, presented here, extend this range to about 3000 Ma, suggesting continuous low-Ti and VLT basaltic magmatism for at least 200 Myr.

Nevertheless, the identification of distinct magmatic episodes in the overall set of basaltic rocks available for study continues to be hampered by the discrepancy of ages obtained for a sample dated by different techniques. This problem is particularly severe in the lunar meteorite samples, leading to a potentially biased picture of the timing of the magmatic activity on the Moon (see Fig. 9a). Therefore, the entire data set of previously published lunar meteorite ages including those from NWA 4734, Dhofar 287A, and NWA 773 clan, was screened in an attempt to filter the most reliable data and reassess information about lunar basaltic magmatism that can be extracted from the available meteorite ages. In addition to the samples investigated in this study, the combined available age data set includes nine stones or clans (KAL 009, MIL 13317, Y-793169, A-881757, EET 96008, the LAP 02204 and 02205; paired stones, MET 01210, NEA 003A, and the group including NWA 032-NWA 479 rocks) with the entire data set representing a total of 57 ages obtained by Lu-Hf, Sm-Nd, Ar-Ar, Rb-Sr, U-Pb, and Pb-Pb methods.

As mentioned earlier, ^40^Ar/^39^Ar measurements are specifically subject to resetting and tend to yield younger ages than those obtained by other methods (Fig. 9a). In addition, whole-rock Ar analyses might include material affected by terrestrial alteration or containing impact melt leading to biased ages (e.g., Fernandes et al. 2003). Therefore, Ar data are often difficult to use when investigating magmatic ages of basaltic meteorites. The reliability of individual Lu-Hf, Sm-Nd, and Rb-Sr ages was assessed on the basis of goodness of the fit for the isochrons determined by MSWD lower than 2 and *P* (probability of the fit) higher than 0.05. In addition, analyses with the calculated uncertainty on the age in excess of more than 3% (i.e., about 100 Ma) were excluded as their uncertainties cover entire periods proposed for duration of low- and high-Ti basalts based on the studies of Apollo mare basalt samples. Also, for Rb-Sr, Lu-Hf, and Sm-Nd isochrons, the data were reprocessed to remove whole-rock fractions as they might include terrestrial material and impact melts that can bias the calculated age. U-Pb or Pb-Pb data were plotted in a ^204^Pb/^206^Pb versus ^207^Pb/^206^Pb diagram to assess the possible presence of terrestrial Pb contamination and, when required, reprocessed to eliminate potential biases related to this contamination.

**Fig. 10:**
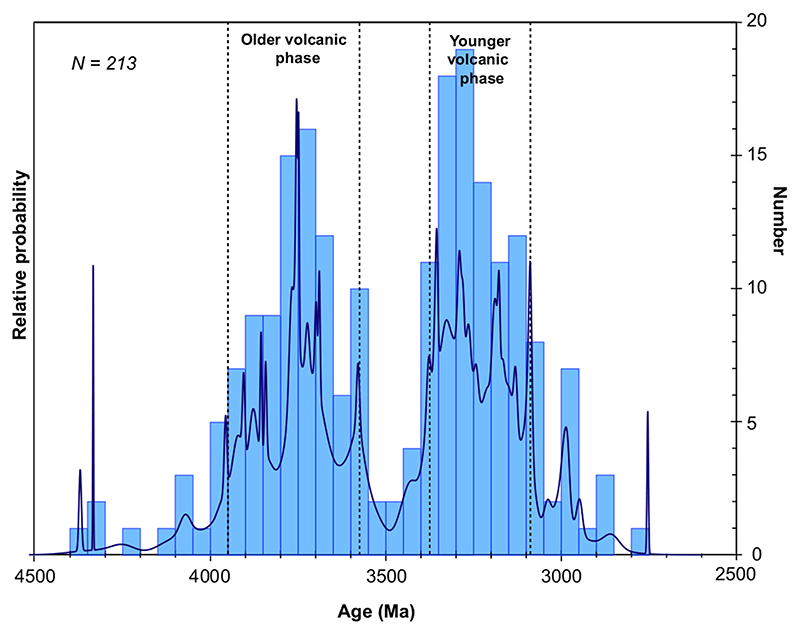
Probability density plot representing the age distribution of Apollo mafic rocks and filtered gabbroic and basaltic lunar meteorite ages filtered data set. The data used to construct this plot are available in supporting information. Same references for the Data as in Fig. 9. Bin size: 50 Ma. (Color figure can be viewed at wileyonlinelibrary.com.)

The updated lunar meteorite age data set includes 11 meteorites and 27 ages (Fig. 9b). The available data appear to cluster between 4300-3800 Ma and 32003000 Ma (Fig. 9b). It should be noted that for each stone in the filtered meteorite age data set, there are no more than three ages, most of them overlapping within uncertainties while the unfiltered data set included up to 10 dates (Fig. 9a). A few samples still display discrepancies between ages obtained by different techniques (KAL 009, A-881757, and LAP 02205-02224 paired stones, Fig. 9b) that are interpreted as either (1) remaining analytical issues or (2) the presence of different rocks of distinct ages within a given stone as identified in NWA 773 clan. The NWA 773 clan is overrepresented owing to the large number of samples included in this clan which have been dated (six in total). By combining the accepted ages from meteorites and Apollo samples as well as basaltic clasts from the Soviet Luna 16 and 20 missions, the resulting data set includes 213 ages representing 95 samples allowing a graphic representation of these data using a density probability diagram to visualize the timing of the mafic magmatic activity on the Moon (Fig. 10). The small number of samples considered here limits this approach as these samples might not be representative of the entire magmatic activity of the Moon. Nevertheless, this does not preclude attempts to review the current available data and use it to make the best interpretation possible.

The density probability plot shows two distinct main episodes of mare basalt magmatism occurred around 3950–3575 Ma and 3375–3075 Ma, separated by a 200 Ma gap. This suggests that the magmatic activity was not continuous and focussed at 3800–3300 Ma as previously interpreted from the crater size–frequency distribution and previous age compilations. The younger volcanic phase (3375–3075 Ma) is dominated by rocks from the low-Ti group while the older phase (3950–3575 Ma) involves the low-Ti, high-Ti, high-Al, and KREEP chemical groups (Fig. 9b). Furthermore, there is a gap of 300 Ma between the end of the low-Ti activity during the older phase and the younger phase (3850–3350 Ma, Fig. 9b).

### Initial Pb Composition of Investigated Lunar Meteorite Samples

As expressed above, initial Pb compositions are estimated from the data points that fall at the top end of the isochron. The confidence in the accuracy of initial Pb determinations increases with the number of points analyzed within the sample clustering at the top end of the isochron.

Regardless, these estimates can only be considered to represent minimum limits for the true values of initial Pb compositions of the samples. The best estimates of ^204^Pb/^206^Pb and ^207^Pb/^206^Pb initial values for samples NWA 2727 and Dhofar 287A are based on one analysis located at the top end of the respective isochrons and are, therefore, unreliable. Values for five other samples analyzed here and shown in Table 1 are estimated using an average of three to five K-feldspar analyses and are assumed to be very close to the true values. However, even these compositions can be underestimated, which could be especially the case for NWA 3170, showing ^204^Pb/^206^Pb and ^207^Pb/^206^Pb initial ratios that are slightly lower than those obtained for two other samples with a similar age. Alternatively, it may also indicate a real range of initial compositions in the suite of rocks represented by the NWA 773 clan of meteorites. Regardless of these limitations in relation to the initial Pb estimates, the highly radiogenic nature of lunar Pb results in a much stronger effect of this underestimation on the ^207^Pb/^206^Pb ratios. The impact on ^204^Pb/^206^Pb is less severe and these ratios can be used to investigate general trends in isotope compositions displayed by different suites of lunar basaltic rocks.

**Fig. 11:**
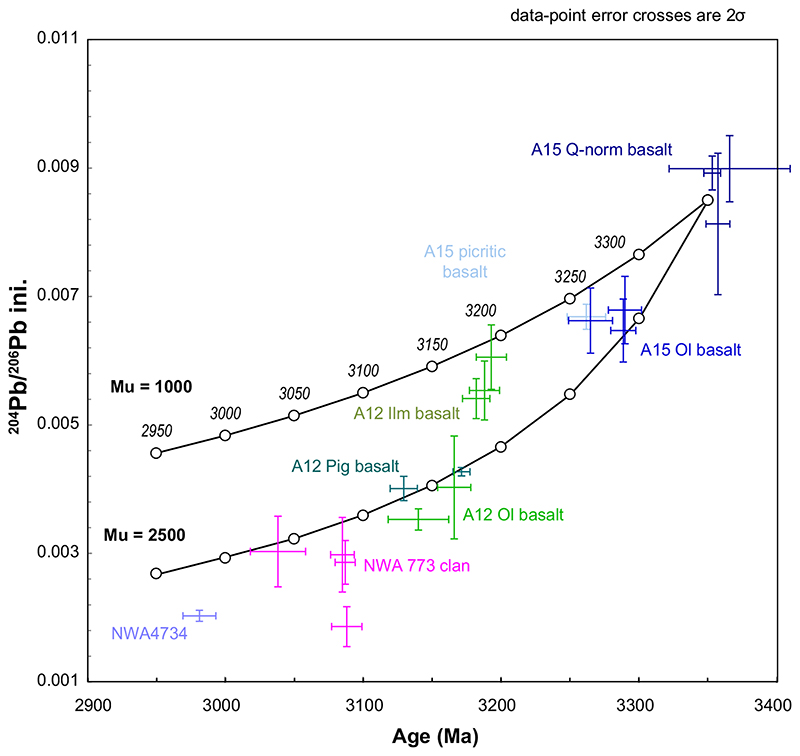
Variation of initial ^204^Pb/^206^Pb versus age (in Ma) for the low-Ti Apollo samples and NWA 4734 and NWA 773 clan meteorites. Also shown are the evolution curves of the ^204^Pb/^206^Pb ratio with age corresponding to μ values of 1000 and 2500. Data for Apollo samples from Snape et al. (2019). Data for lunar meteorites from this work. (Color figure can be viewed at wileyonlinelibrary.com.)

By combining meteorite data obtained here with previously published results obtained for low-Ti mare basalts from Apollo 12 and Apollo 15 landing sites (Snape et al. 2018, 2019) on a ^204^Pb/^206^Pb versus age diagram (Fig. 11), a systematic decrease of this ratio is observed as the basalts are younger. The samples from the NWA 773 clan that are considered to show typical KREEP chemical signatures are located at the end of this trend (Fig. 11). This trend cannot represent the evolution of a single homogeneous source between about 3350 Ma (age of Apollo 15 basalts) and approximately 3050 Ma (age of the meteorite samples dated here). Indeed, the source must have a μ value of at least 2500. This value is inconsistent with the previous estimates for the sources of low-Ti mare basalts and more compatible with the KREEP reservoir, source of the Apollo 15 KREEP basalts (e.g., Snape et al. 2019). In addition, low and very low-Ti basalts are probably derived from a heterogeneous source (e.g. Neal and Taylor 1992; Elardo et al. 2014) as illustrated by the different initial Pb ratios we document in the NWA 773 clan. It should be noted that NWA 4734, which has enriched chemical characteristics but not considered as typical of KREEP basalts similar to those collected by the Apollo missions (Elardo et al. 2014), does not fit this trend. Consequently, the most likely explanation of the age-related chemical trend observed in the meteorite and Apollo low and very low-Ti basalts is a progressive contribution of a component with a KREEP-like signature, resulting in the younger basalts showing a stronger KREEP-like affinity.

## Conclusions

Using the SIMS in situ Pb-Pb dating technique, we obtained robust isochron ages for NWA 4734 and Dhofar 287A. Four samples (NWA 2727, NWA 773, NWA 2977, NWA 3170) of six of the NWA 773 clan yielded statistically valid isochron ages that are identical within uncertainties. The average age of these four samples is 3086.1 ± 4.8 Ma (2σ, MSWD = 0.16, P = 0.92). The gabbroic sample NWA 3333 yielded a slightly younger robust isochron date of 3038 ± 20 Ma suggesting that two magmatic events are recorded in the stones of the NWA 773 clan: an older one at 3086 Ma and a younger one at 3038 Ma.

Our new ages tend to be younger than the U-Pb and Pb-Pb ages but older than most of the whole-rock ^40^Ar-^39^Ar ages previously published. This discrepancy is related to differences in approach, used by different studies to account for the terrestrial Pb contamination for the former ages and resetting and possible inclusion of impact melt for the latter ages.

All of our new ages record magmatic events at the younger end of those recorded by the Apollo samples (approximately 3950–3150 Ma; e.g., Nyquist and Shih 1992; Shearer and Papike 1999; Joy and Arai 2013; Snape et al. 2019; Tartèse et al. 2019), providing clear evidence of significant magmatic activity on the Moon around 3100–3000 Ma. Combining the filtered lunar meteorite age data set with those of the Apollo mafic rocks shows that (1) the mafic magmatic activity on the Moon was not continuous and (2) two main episodes of mare basalt magmatism occurred around 3950–3575 Ma and 3375–3075 Ma instead of one continuous phase between 3900 and 3200 Ma as previous suggested (e.g., Hiesinger et al. 2003, 2011).

The time-related evolution of the Pb initial ratios determined in the low-Ti basalts formed between ~3400 and ~3100 Ma suggests a progressive contribution of a KREEP-like component in the chemical characteristics of these rocks.

## Supplementary Material

Annex

Fig. A1-9

Lunar compilation

Pb data

Standard

Supporting Information

## Figures and Tables

**Table 1:** Summary of ages and Pb initial ratios for the lunar meteorites of the NWA 773 clan, Dhofar 287 and NWA 4734.

Sample name	Texture	Chemical group	Age (Ma)	±2σ abs	(^207^Pb/^206^Pb)_ini_	± 2σ abs	(^204^Pb/^206^Pb)_ini_	±2σ abs	Notes
NWA 4734	Basalt (coarsegrained)	Low-Ti	2981	12	0.8425	0.01	0.002028	9E-05	Three weighted average values for initials calculation
Dhofar 287	Basalt (coarsegrained)	Low-Ti	3208	22	*0.9669*	*0.01*	*0.00274*	*0.0004*	Initial = highest measured value
NWA 2700	Basaltic (fine-grained)	Very low-Ti	2871	300	-	-	-	-	
NWA 2727	Basaltic (fine-grained)	Very low-Ti	3081	21	*0.7857*	*0.01*	*0.01348*	*0.0009*	Five points regression. initial = highest measured value
NWA 773	Gabbroic	Very low-Ti	3087	7.3	*1.124*	*0.01*	0.00286	0.0003	^207^Pb/^206^Pb = highest measured value. Four data weighted average for ^204^Pb/^206^Pb
NWA 2977	Gabbroic	Very low-Ti	3085	8.6	1.108	0.01	0.00298	0.0006	Weighted average for initials calculation (*N* = 4)
NWA 3170	Gabbroic	Very low-Ti	3088	11	1.105	0.01	0.00186	0.0003	Weighted average for initials calculation (*N* = 5)
NWA 3333	Gabbroic	Very low-Ti	3038	20	1.122	0.02	0.00303	0.0006	Two data weighted average for ^207^Pb/^206^Pb. Three data weighted average for ^204^Pb/^206^Pb

Data in italic are considered as unreliable.
