# Supplementary material for: Pb-Pb ages and initial Pb isotopic composition of lunar meteorites: NWA 773 clan, NWA 4734, and Dhofar 287
Source: Meteorit Planet Sci. Author manuscript; Available in PMC 2021 Aug 9. (PMC7611475; doi:10.1111/maps.13547)
Supplement: Annex [file EMS131186-supplement-Annex.docx]

**Pb-Pb ages and Pb initial isotopic composition of lunar meteorites: NWA 773 clan, NWA 4734 and Dhofar 287**

By Merle et al.

**Supplementary material**

**List of figures:**

Figure A1: Calculation of weighted average ^207^Pb/^206^Pb and ^204^Pb/^206^Pb initial ratios for NWA 4734.

Figure A2: Calculation of weighted average ^207^Pb/^206^Pb and ^204^Pb/^206^Pb initial ratios for NWA 773.

Figure A3: Calculation of weighted average ^207^Pb/^206^Pb and ^204^Pb/^206^Pb initial ratios for NWA 2977.

Figure A4: Calculation of weighted average ^207^Pb/^206^Pb and ^204^Pb/^206^Pb initial ratios for NWA 3170.

Figure A5: Calculation of weighted average ^207^Pb/^206^Pb and ^204^Pb/^206^Pb initial ratios for NWA 3333.

Figure A6: ^204^Pb/^206^Pb plot for assessing the possible contribution of terrestrial Pb in the previously published data for NWA 4734. Our new data are also shown for comparison. The previously published data have been reprocessed and age recalculated using the same approach used for our new data.

Figure A7: ^204^Pb/^206^Pb plot for assessing the possible contribution of terrestrial Pb in the previously published data for Dhofar 287. Our new data are also shown for comparison. The previously published data have been reprocessed and age recalculated using the same approach used for our new data.

Figure A8: ^204^Pb/^206^Pb plot for assessing the possible contribution of terrestrial Pb in the previously published data for NWA 2977. Our new data are also shown for comparison. The previously published data have been reprocessed and age recalculated using the same approach used for our new data.

Figure A9: Previously published and new data for NWA 773 shown in conventional and reverse Concordia diagrams as well as in a ^204^Pb/^206^Pb plot to assess the possible involvement of terrestrial Pb contamination.

**Datasets:**

- New Pb-Pb data for NWA 773, NWA 3333, NWA 3170, NWA 2727, NWA 2700, NWA 2977, NWA 3734 and Dhofar 287.
- Standards analysed during the different analytical sessions. Also provided, the correction factors.
- Lunar mafic meteorites dataset (unfiltered and filtered).
