# Supplementary material for: Pb-Pb ages and initial Pb isotopic composition of lunar meteorites: NWA 773 clan, NWA 4734, and Dhofar 287
Source: Meteorit Planet Sci. Author manuscript; Available in PMC 2021 Aug 9. (PMC7611475; doi:10.1111/maps.13547)
Supplement: Fig. A1-9 [file EMS131186-supplement-Fig__A1_9.pdf]

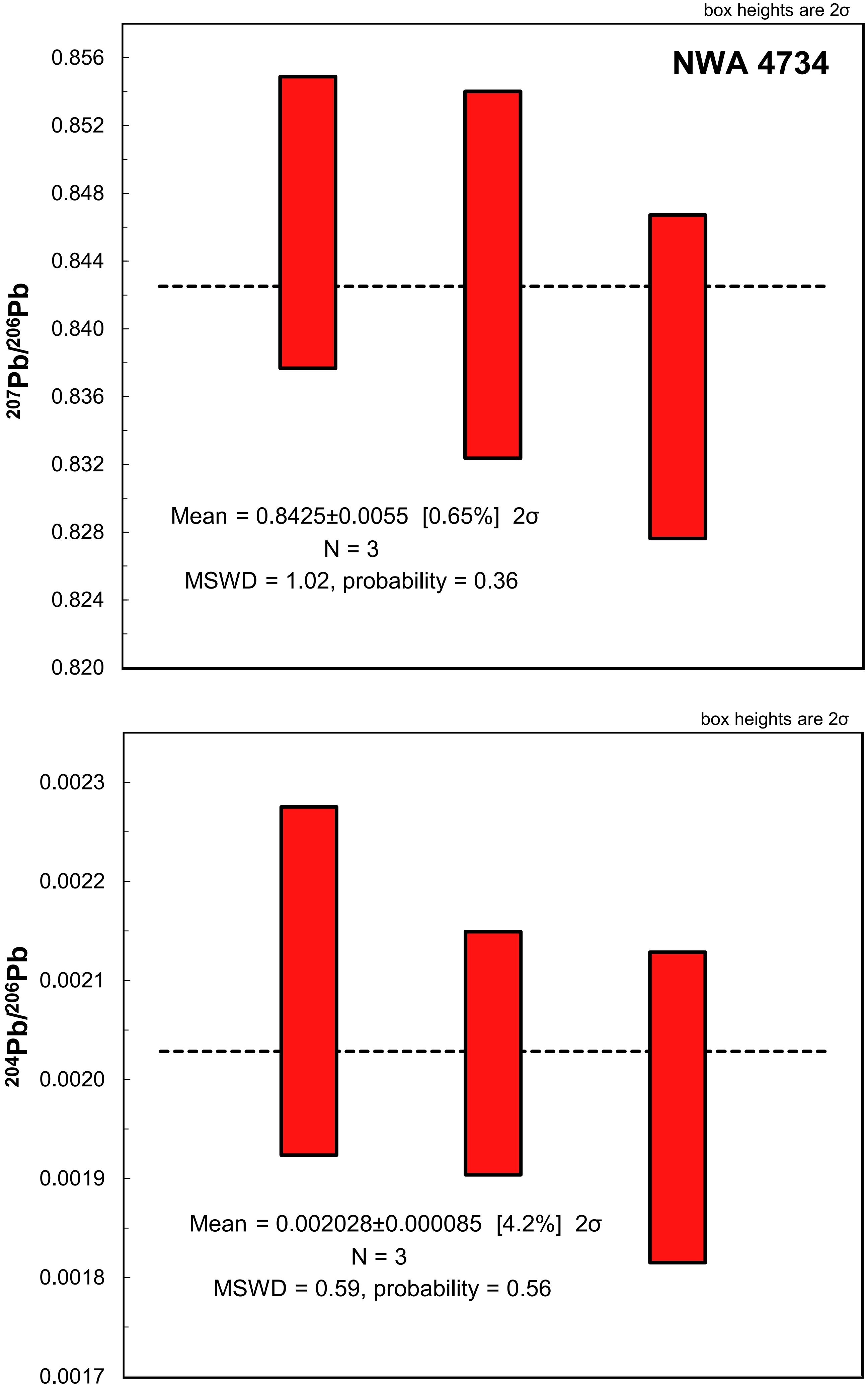
box heights are 2σ
NWA 4734
0.856
0.852
0.848
0.844
0.840
0.836
0.832
0.828
0.824
0.820
207Pb/206Pb
Mean = 0.8425±0.0055 [0.65%] 2σ
N = 3
MSWD = 1.02, probability = 0.36
box heights are 2σ
0.0023
0.0022
0.0021
0.0020
0.0019
0.0018
0.0017
204Pb/206Pb
Mean = 0.002028±0.000085 [4.2%] 2σ
N = 3
MSWD = 0.59, probability = 0.56

Figure A1

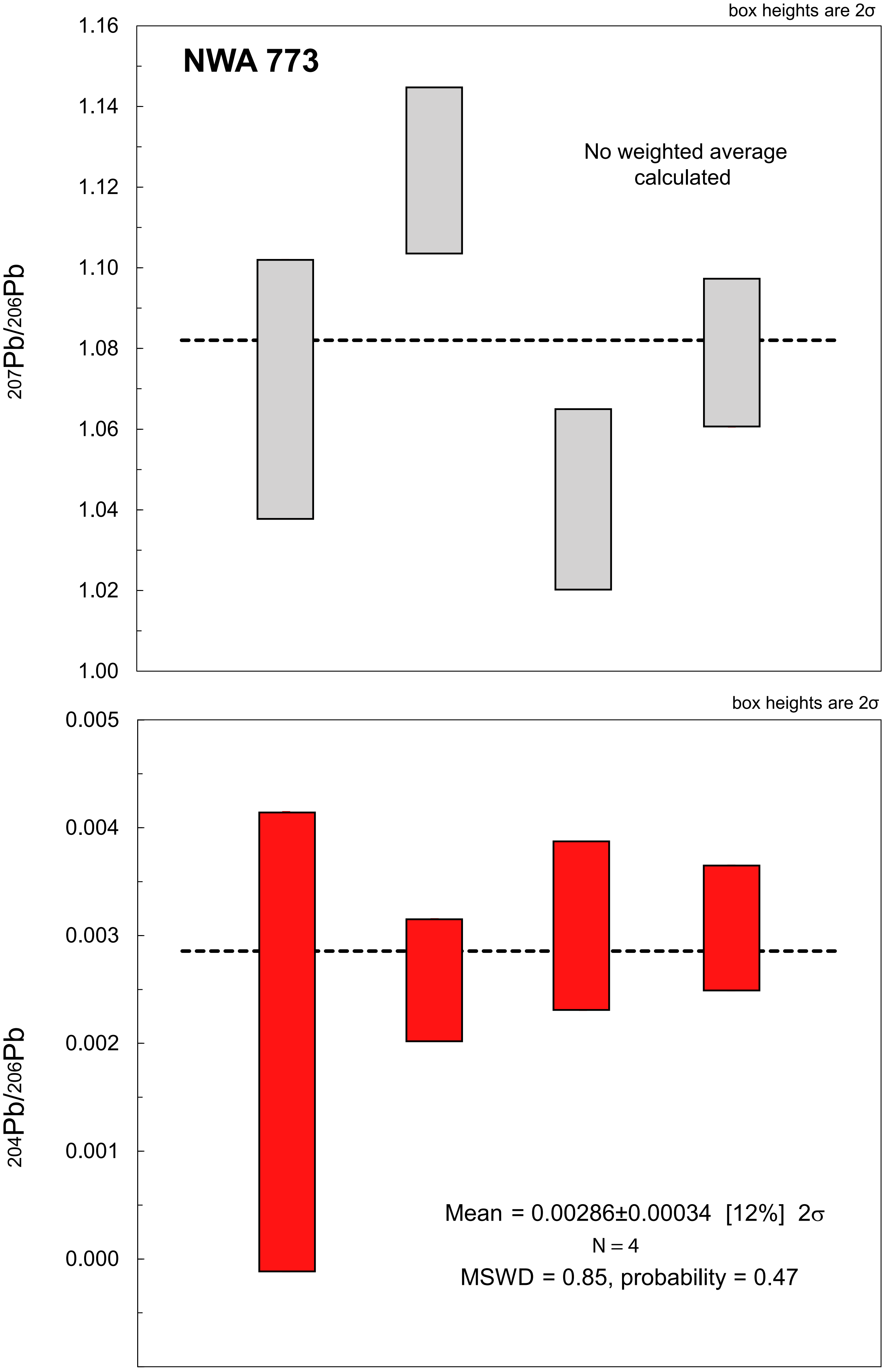
box heights are 2σ
NWA 773
No weighted average calculated
207Pb/206Pb
1.16
1.14
1.12
1.10
1.08
1.06
1.04
1.02
1.00
box heights are 2σ
204Pb/206Pb
0.005
0.004
0.003
0.002
0.001
0.000
Mean = 0.00286±0.00034 [12%] 2σ
N = 4
MSWD = 0.85, probability = 0.47

Figure A2

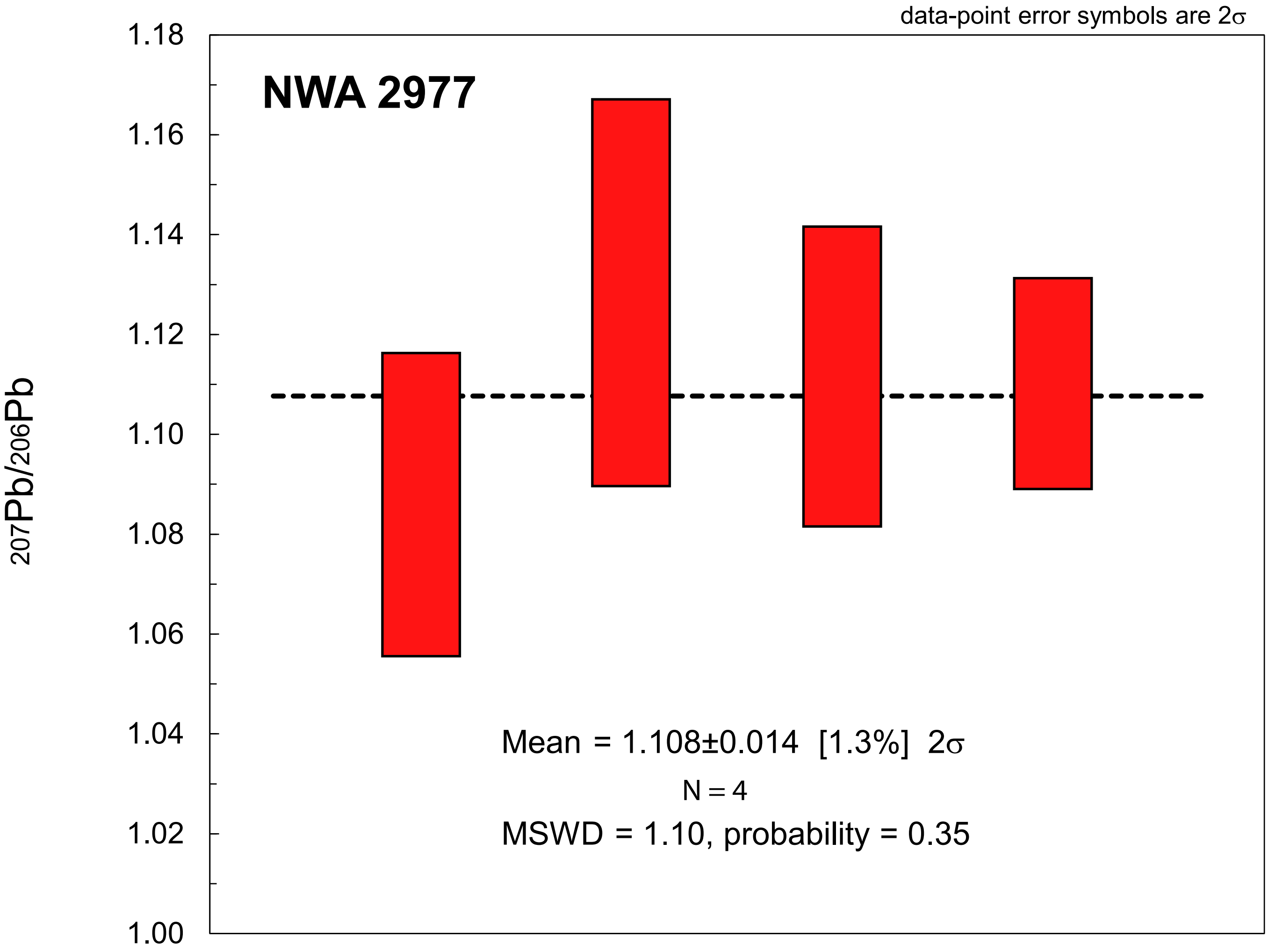

data-point error symbols are 2σ
NWA 2977
1.18
1.16
1.14
1.12
1.10
1.08
1.06
1.04
1.02
1.00
207Pb/206Pb
Mean = 1.108±0.014 [1.3%] 2σ
N = 4
MSWD = 1.10, probability = 0.35

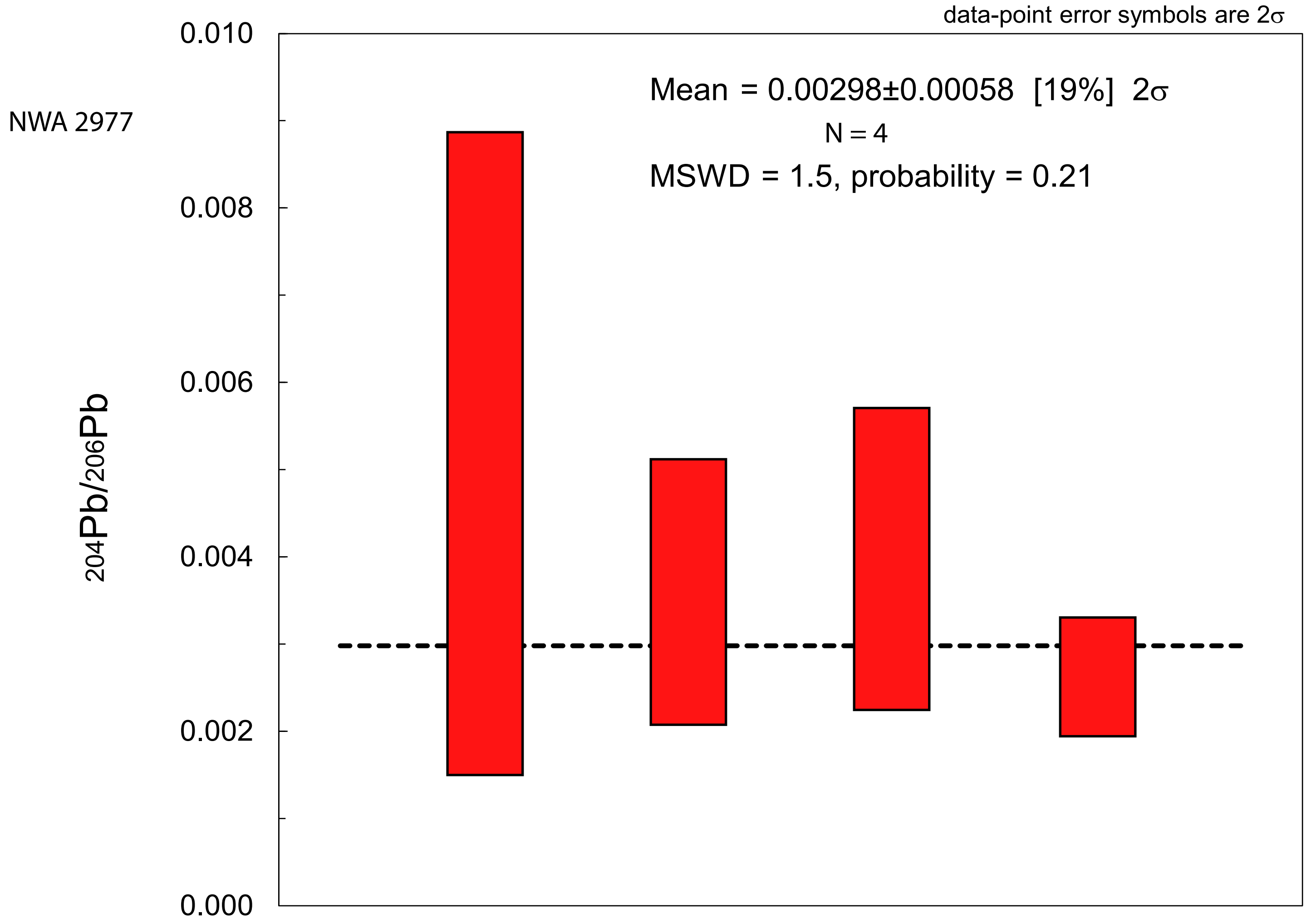

data-point error symbols are 2σ
NWA 2977
0.010
0.008
0.006
0.004
0.002
0.000
204Pb/206Pb
Mean = 0.00298±0.00058 [19%] 2σ
N = 4
MSWD = 1.5, probability = 0.21

Figure A3

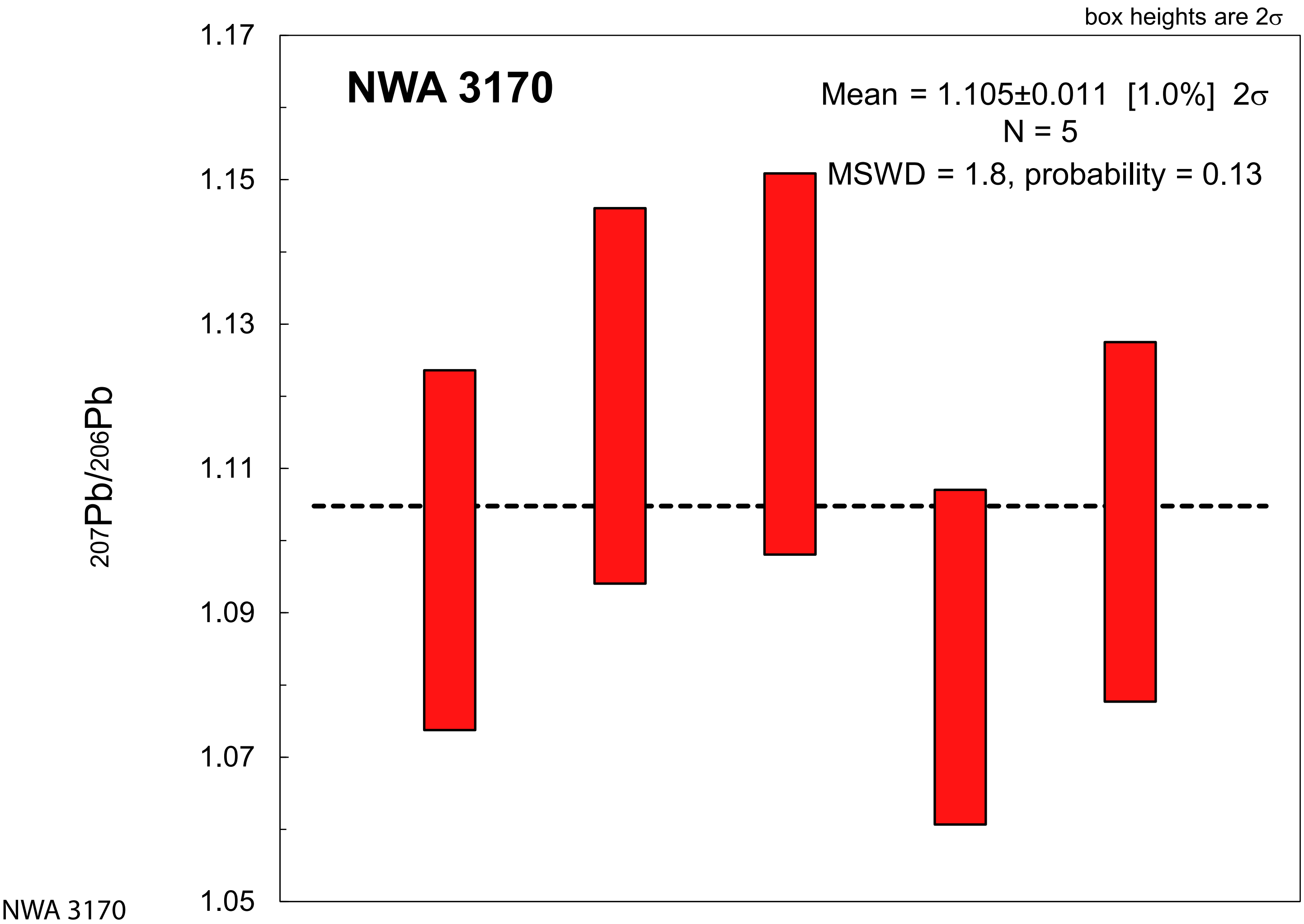

NWA 3170

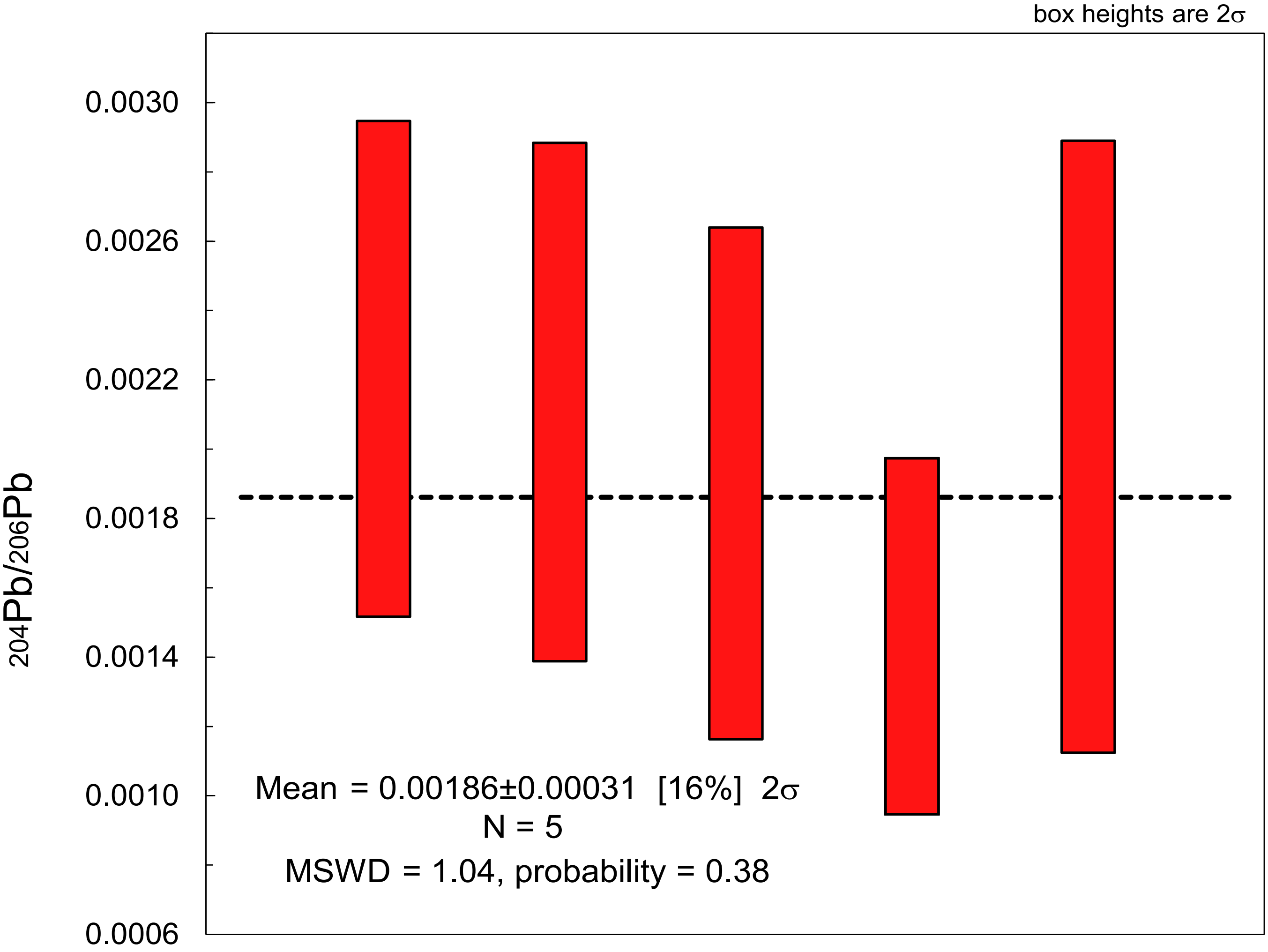

Figure A4

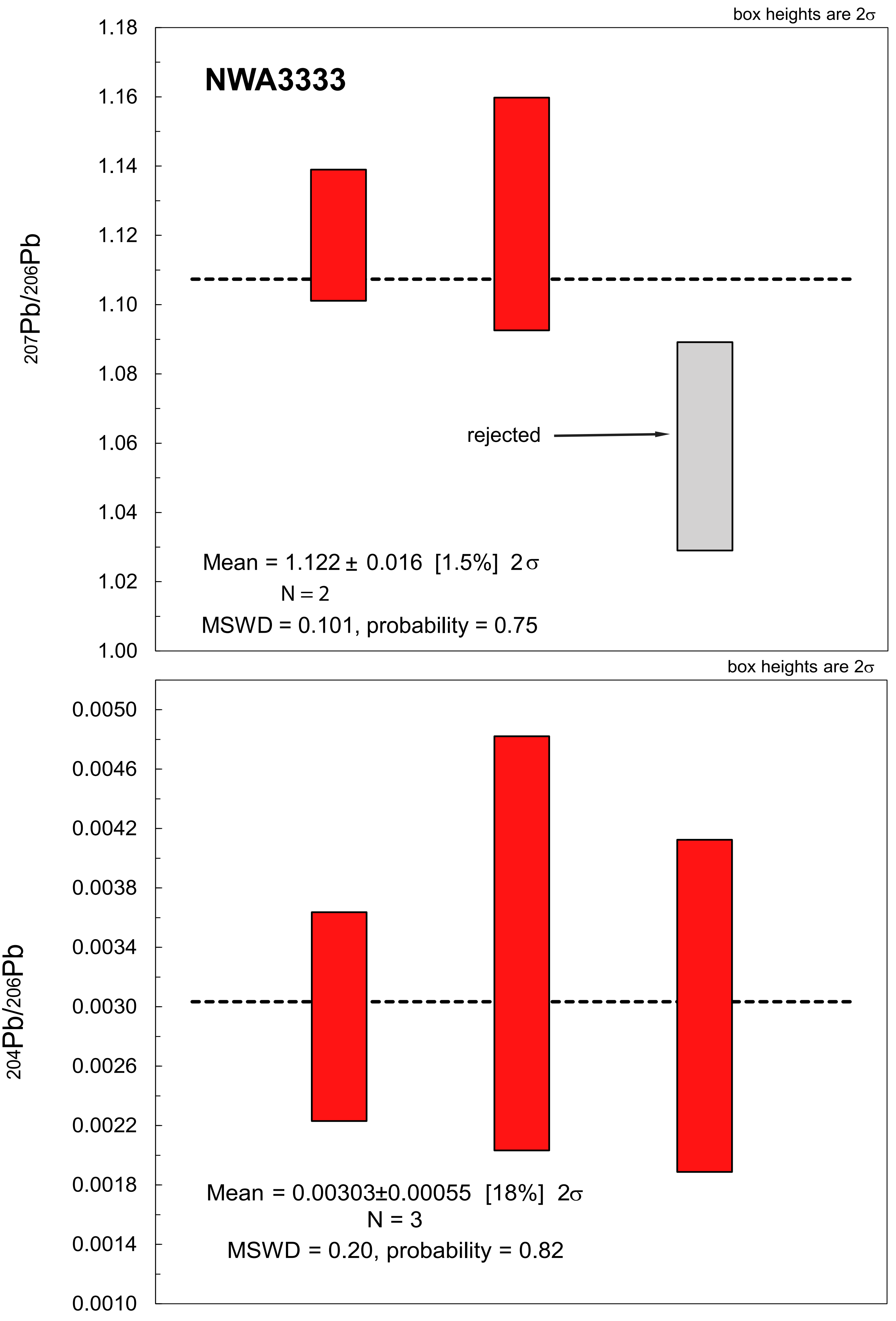

box heights are 2σ
NWA3333
1.18
1.16
1.14
1.12
1.10
1.08
1.06
1.04
1.02
1.00
207Pb/206Pb
rejected
Mean = 1.122 ± 0.016 [1.5%] 2σ
N = 2
MSWD = 0.101, probability = 0.75
box heights are 2σ
0.0050
0.0046
0.0042
0.0038
0.0034
0.0030
0.0026
0.0022
0.0018
0.0014
0.0010
204Pb/206Pb
Mean = 0.00303±0.00055 [18%] 2σ
N = 3
MSWD = 0.20, probability = 0.82

Figure A5

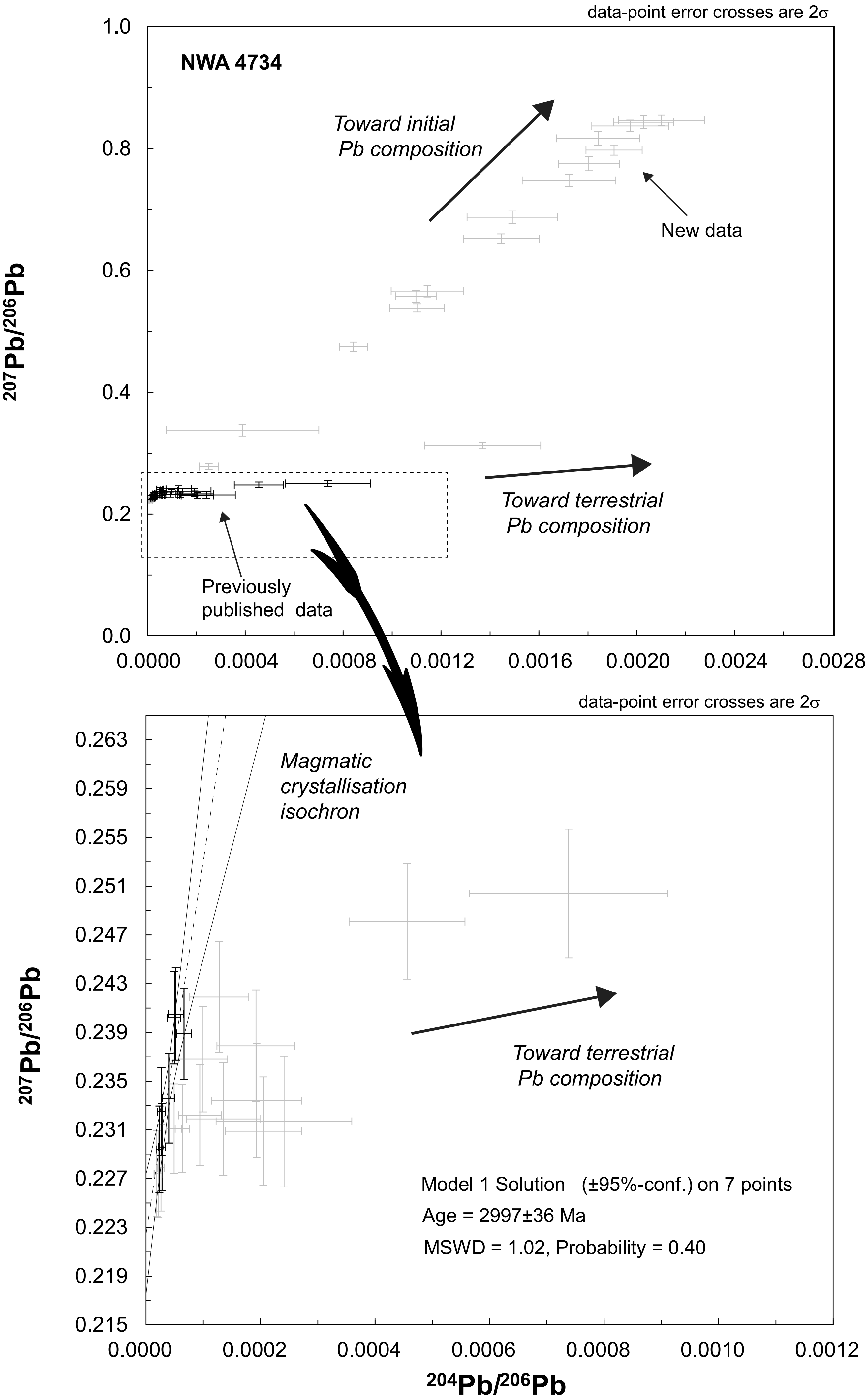

data-point error crosses are 2σ
NWA 4734
Toward initial Pb composition
New data
Toward terrestrial Pb composition
Previously published data
207Pb/206Pb
1.0
0.8
0.6
0.4
0.2
0.0
0.0000
0.0004
0.0008
0.0012
0.0016
0.0020
0.0024
0.0028
data-point error crosses are 2σ
Magmatic crystallisation isochron
Toward terrestrial Pb composition
Model 1 Solution (±95%-conf.) on 7 points
Age = 2997±36 Ma
MSWD = 1.02, Probability = 0.40
207Pb/206Pb
0.263
0.259
0.255
0.251
0.247
0.243
0.239
0.235
0.231
0.227
0.223
0.219
0.215
0.0000
0.0002
0.0004
0.0006
0.0008
0.0010
0.0012
204Pb/206Pb

Figure A6

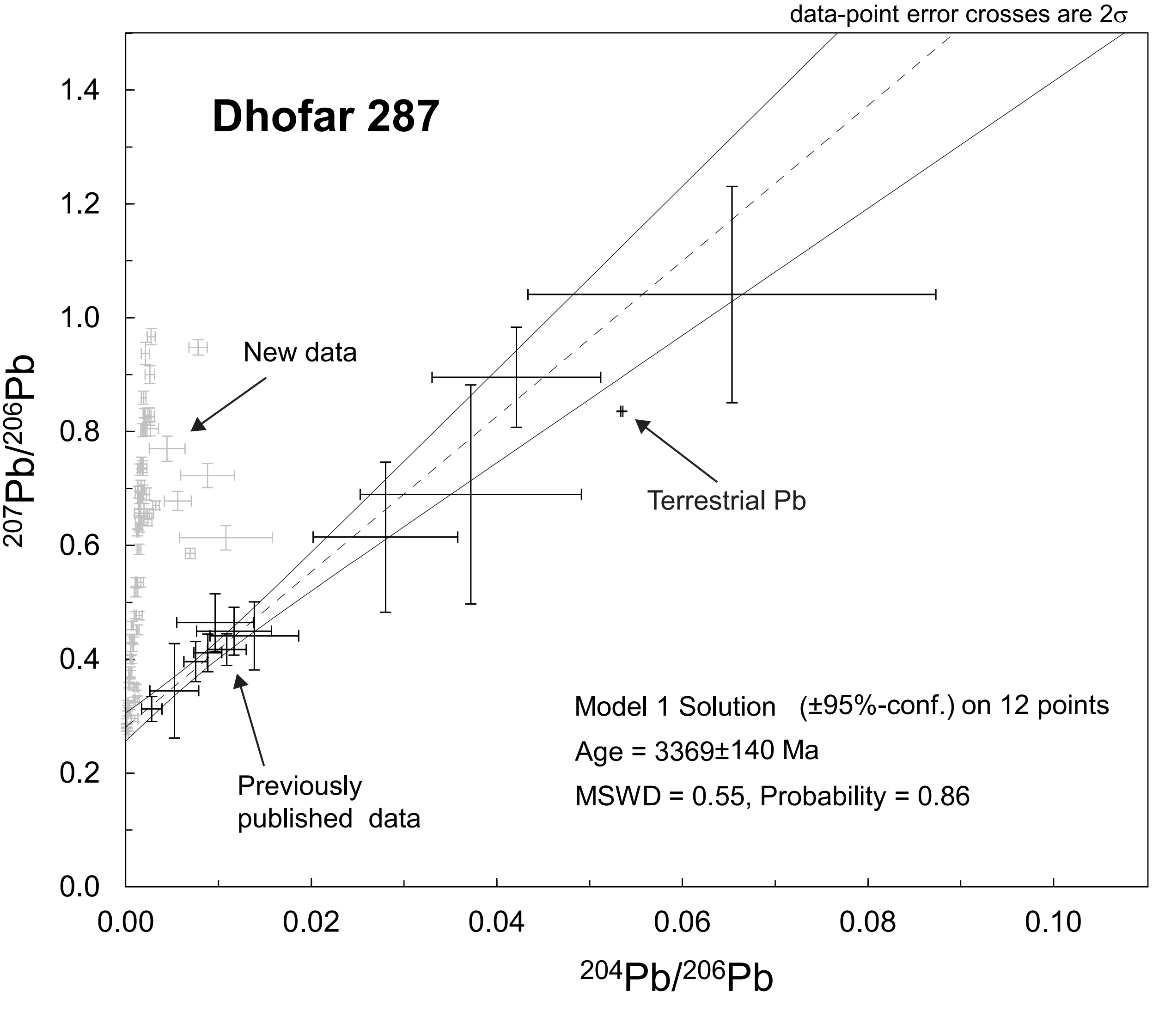
data-point error crosses are 2σ
Dhofar 287
New data
Terrestrial Pb
Previously published data
Model 1 Solution (±95%-conf.) on 12 points
Age = 3369±140 Ma
MSWD = 0.55, Probability = 0.86
207Pb/206Pb
204Pb/206Pb
0.0
0.2
0.4
0.6
0.8
1.0
1.2
1.4
0.00
0.02
0.04
0.06
0.08
0.10

Figure A7

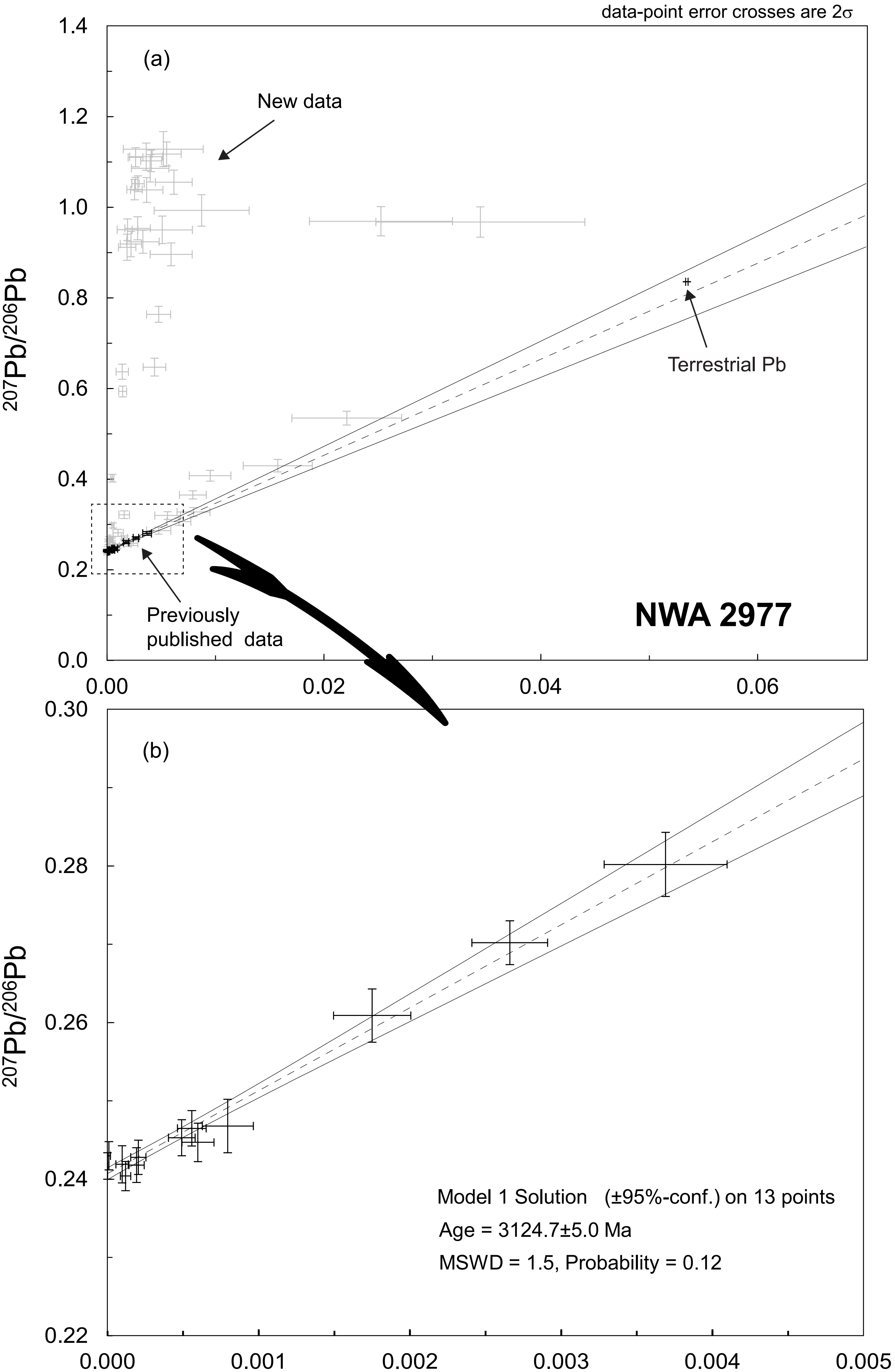

data-point error crosses are 2σ
(a)
New data
Terrestrial Pb
Previously published data
NWA 2977
(b)
Model 1 Solution (±95%-conf.) on 13 points
Age = 3124.7±5.0 Ma
MSWD = 1.5, Probability = 0.12
207Pb/206Pb
204Pb/206Pb

Figure A8

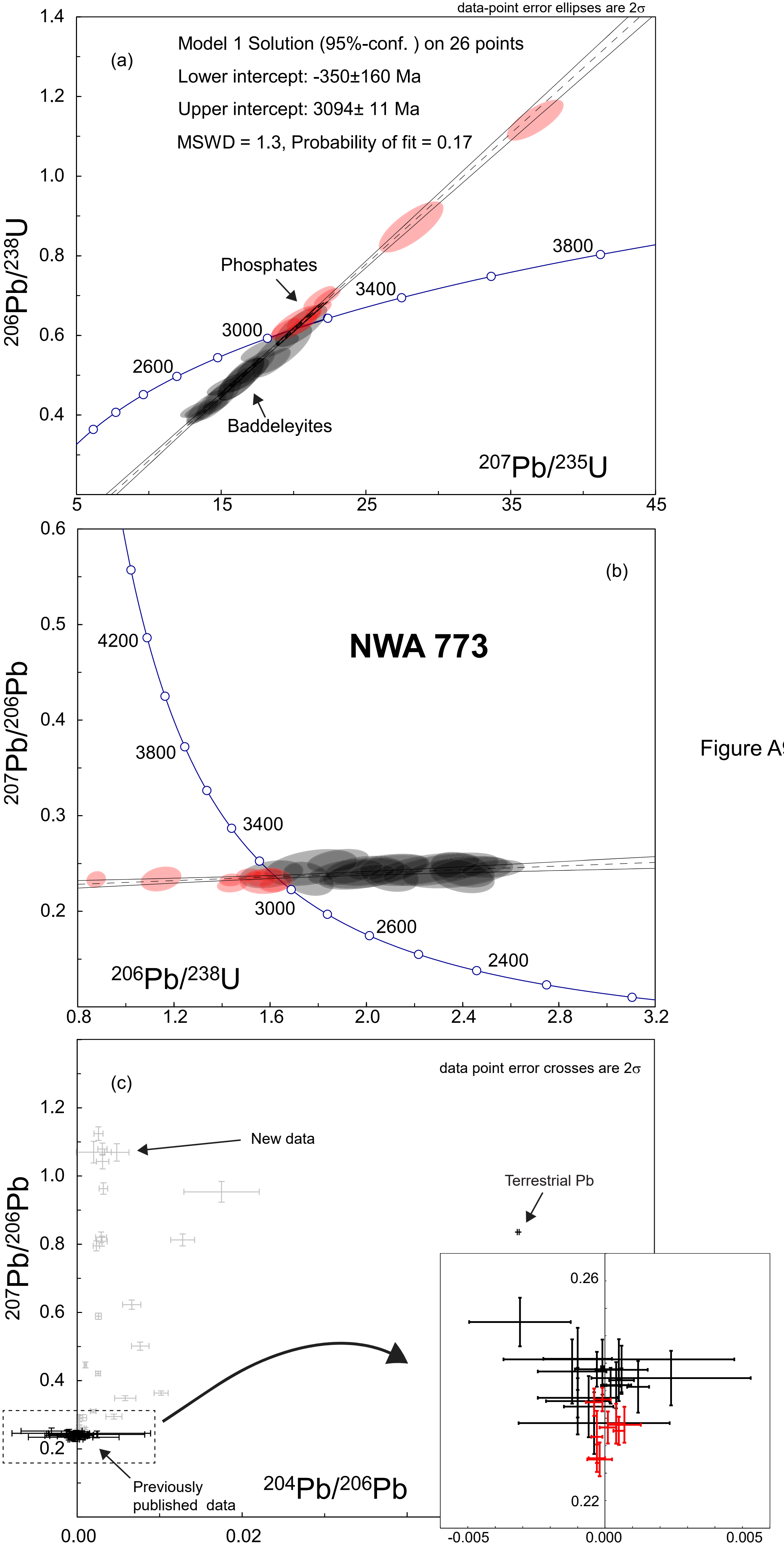
data-point error ellipses are 2σ
(a)
Model 1 Solution (95%-conf.) on 26 points
Lower intercept: -350±160 Ma
Upper intercept: 3094± 11 Ma
MSWD = 1.3, Probability of fit = 0.17
Phosphates
Baddeleyites
3800
3400
3000
2600
$^{206}Pb/^{238}U$
$^{207}Pb/^{235}U$
1.4
1.2
1.0
0.8
0.6
0.4
5
15
25
35
45
(b)
NWA 773
4200
3800
3400
3000
2600
2400
$^{207}Pb/^{206}Pb$
$^{206}Pb/^{238}U$
0.6
0.5
0.4
0.3
0.2
0.8
1.2
1.6
2.0
2.4
2.8
3.2
(c)
data point error crosses are 2σ
New data
Terrestrial Pb
Previously published data
$^{207}Pb/^{206}Pb$
$^{204}Pb/^{206}Pb$
1.2
1.0
0.8
0.6
0.4
0.2
0.00
0.02
0.26
0.22
-0.005
0.000
0.005

Figure A9
