# Supplementary material for: Pb-Pb ages and initial Pb isotopic composition of lunar meteorites: NWA 773 clan, NWA 4734, and Dhofar 287
Source: Meteorit Planet Sci. Author manuscript; Available in PMC 2021 Aug 9. (PMC7611475; doi:10.1111/maps.13547)
Supplement: Supporting Information [file EMS131186-supplement-Supporting_Information.pdf]

SUPPORTING INFORMATION

Additional supporting information may be found in the online version of this article.

Fig. A1. Calculation of weighted average $^{207}Pb/^{206}Pb$ and $^{204}Pb/^{206}Pb$ initial ratios for NWA 4734.

Fig. A2. Calculation of weighted average $^{207}Pb/^{206}Pb$ and $^{204}Pb/^{206}Pb$ initial ratios for NWA 773.

Fig. A3. Calculation of weighted average $^{207}Pb/^{206}Pb$ and $^{204}Pb/^{206}Pb$ initial ratios for NWA 2977.

Fig. A4. Calculation of weighted average $^{207}Pb/^{206}Pb$ and $^{204}Pb/^{206}Pb$ initial ratios for NWA 3170.

Fig. A5. Calculation of weighted average $^{207}Pb/^{206}Pb$ and $^{204}Pb/^{206}Pb$ initial ratios for NWA 3333.

Fig. A6. $^{204}Pb/^{206}Pb$ plot for assessing the possible contribution of terrestrial Pb in the previously published data for NWA 4734. Our new data are also shown for comparison. The previously published data have been reprocessed and age recalculated using the same approach used for our new data.

Fig. A7. $^{204}Pb/^{206}Pb$ plot for assessing the possible contribution of terrestrial Pb in the previously published data for Dhofar 287. Our new data are also shown for comparison. The previously published data have been reprocessed and age recalculated using the same approach used for our new data.

Fig. A8. $^{204}Pb/^{206}Pb$ plot for assessing the possible contribution of terrestrial Pb in the previously published data for NWA 2977. Our new data are also shown for comparison. The previously published data have been reprocessed and age recalculated using the same approach used for our new data.

Fig. A9. Previously published and new data for NWA 773 shown in conventional and reverse Concordia diagrams as well as in a $^{204}Pb/^{206}Pb$ plot to assess the possible involvement of terrestrial Pb contamination.

Data Set S1 New Pb-Pb data for NWA 773, NWA 3333, NWA 3170, NWA 2727, NWA 2700, NWA 2977, NWA 3734, and Dhofar 287.

Data Set S2. Standards analyzed during the different analytical sessions. Also provided, the correction factors.

Data Set S3. Lunar mafic meteorites data set (unfiltered and filtered).
